# Metabolic profiling of synovial fluid in human temporomandibular joint osteoarthritis

**DOI:** 10.3389/fimmu.2024.1335181

**Published:** 2024-03-11

**Authors:** Dahe Zhang, Yuxin Zhang, Simo Xia, Pei Shen, Chi Yang

**Affiliations:** ^1^ Department of Oral Surgery, Shanghai Ninth People’s Hospital, Shanghai Jiao Tong University School of Medicine, Shanghai, China; ^2^ College of Stomatology, Shanghai Jiao Tong University, Shanghai, China; ^3^ National Center for Stomatology, Shanghai, China; ^4^ National Clinical Research Center for Oral Diseases, Shanghai, China; ^5^ Shanghai Key Laboratory of Stomatology, Shanghai, China; ^6^ Shanghai Research Institute of Stomatology, Shanghai, China; ^7^ Research Unit of Oral and Maxillofacial Regenerative Medicine, Chinese Academy of Medical Sciences, Shanghai, China

**Keywords:** metabolome, bone resorption, condyle, biomarkers, pain, metabolism

## Abstract

**Introduction:**

Temporomandibular joint (TMJ) osteoarthritis (OA) is a common TMJ degenerative disease with an unclear mechanism. Synovial fluid (SF), an important component of TMJ, contains various proteins and metabolites that may directly contribute to OA. The present study aimed to investigate the influence of SF in TMJOA at the metabolite level.

**Methods:**

Untargeted and widely targeted metabolic profiling were employed to identify metabolic changes in SF of 90 patients with different TMJOA grades according to TMJ magnetic resonance imaging.

**Results:**

A total 1498 metabolites were detected. Most of the metabolites were amino acids and associated metabolites, benzene and substituted derivatives, and lipids. Among patients with mild, moderate and severe TMJOA, 164 gradually increasing and 176 gradually decreasing metabolites were identified, indicating that biosynthesis of cofactors, choline metabolism, mineral absorption and selenocompound metabolism are closely related to TMJOA grade. Combined metabolomics and clinical examination revealed 37 upregulated metabolites and 16 downregulated metabolites in patients with pain, of which 19 and 26 metabolites were positively and negatively correlated, respectively, with maximum interincisal opening. A model was constructed to diagnose TMJOA grade and nine biomarkers were identified. The identified metabolites are key to exploring the mechanism of TMJOA.

**Discussion:**

In the present study, a metabolic profile was constructed and assessed using a much larger number of human SF samples from patients with TMJOA, and a model was established to contribute to the diagnosis of TMJOA grade. The findings expand our knowledge of metabolites in human SF of TMJOA patients, and provide an important basis for further research on the pathogenesis and treatment of TMJOA.

## Introduction

Temporomandibular joint (TMJ) osteoarthritis (OA) is a common TMJ degenerative disease but the mechanism remains unclear (1). Synovitis, condylar cartilage degeneration and subchondral bone remodeling are the main characteristics of TMJOA (2). Thus, due to the irreplaceable role of condylar cartilage in mandibular development, TMJOA is receiving increasing attention in adolescents and young adults (3). The destruction of condylar cartilage may lead to dentofacial deformities or other dysfunctions, and maintaining condylar cartilage homeostasis is crucial for treating TMJOA (4). Notably, TMJOA and cartilage homeostasis can be influenced directly by the surrounding structures and components, especially the synovial fluid (SF) (4, 5). In our previous work, a proteomic profile of SF samples from patients with anterior disc displacement (ADD)-related TMJOA was constructed, and provided an insight into the pathogenesis of TMJOA at the protein level (5).

In addition to proteins, the roles of metabolites are also receiving increasing attention in TMJOA, and they may be related to inflammation and pain (6, 7). Regarding clinical applications, metabolite biomarkers have been reported in knee OA but they require further validation (8). Several studies have established metabolite profiles of cartilage (9), subchondral bone (10), synovial fluid (11), serum (12), and urine (13) and explored the mechanism of OA. The roles of some important metabolic pathways in OA have been explored including lipid metabolism and glycolytic pathways (8, 14). However, studies involving large numbers of samples exploring metabolite changes in TMJOA are lacking.

In the present study we established a metabolic profile of SF samples from a much larger number of TMJOA patients, and investigated the pathogenesis of TMJOA at the metabolite level. Based on radiological and clinical information, the results provide potential biomarkers for clinical diagnosis or therapeutic targets, and the findings provide a resource for further investigating the mechanism of TMJOA at the metabolite level.

## Methods

### Ethical approval

This retrospective study conformed to the principles of the Declaration of Helsinki and was approved by the Human Research Ethics Committee of Shanghai Ninth People’s Hospital, Shanghai Jiao Tong University School of Medicine (approval no. SH9H-2020-T7-1). Written informed consent was obtained from all participants and their guardians.

### Patients, clinical information, and samples

**Figure 1:**
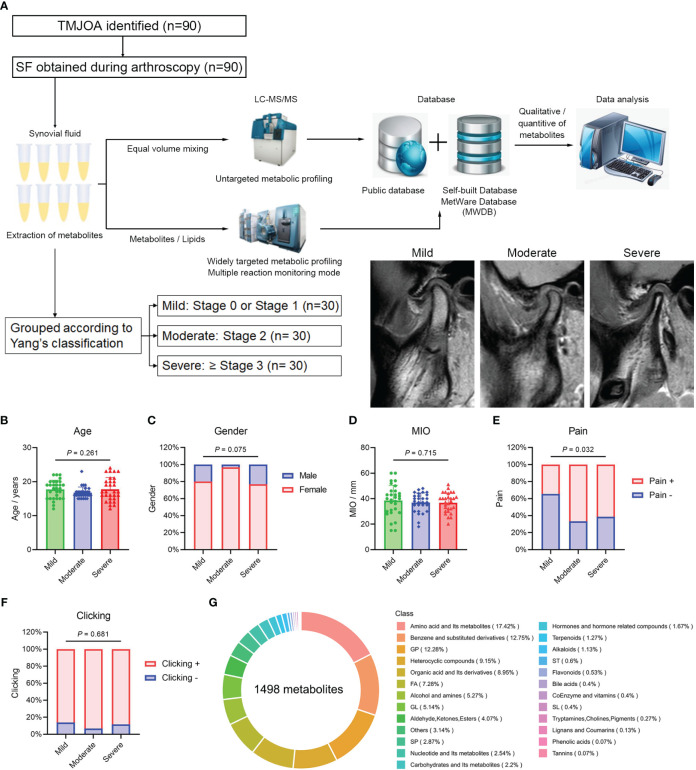
Overview of metabolic profiling. **(A)** Flowchart of the metabolic profiling process and patient grouping. Age **(B)**, gender **(C)**, MIO **(D)**, pain **(E)** and joint clicking **(F)** in Mild, Moderate, and Severe groups. **(G)** Overview and types of identified metabolites. MIO, maximum interincisal opening; GP, glycerophospholipids; FA, fatty acids; GL, glycerolipids; SP, sphingolipids; ST, sterol lipids; SL, saccharolipids.

Patients, aged 10−25 years and diagnosed with ADD-related TMJOA between January 2018 and January 2020 at Shanghai Ninth People’s Hospital, were recruited. Clinical information, including age, gender, maximum interincisal opening (MIO), pain and clicking, were collected. MIO was evaluated by one professional doctor (C.Y) using a conventional ruler. The assessment of the pain was performed by a patient-reported questionnaire. Clicking was examined by one professional doctor (C.Y). According to the TMJ-ADD treatment protocol (15), patients with failed conservative treatments were recommended to undergo arthroscopic discopexy. Patients who agreed to undergo the surgery and provided informed consent were asked to undergo TMJ magnetic resonance imaging (MRI) 1 week before surgery. According to the TMJ MRI examination, patients were divided into three groups following Yang’s classification (16). Specifically, mild or localized resorption without loss of height was defined as mild OA; moderate resorption with reduced height was defined as moderate OA; and a small condyle, significant resorption with loss of cortical bone integrity, or complete condylar resorption was defined as severe OA (**Figure 1A**). At the beginning of the surgery, after a local anesthetic, 1 mL normal saline was injected into the upper TMJ space over 30 s, and this procedure was repeated five times to obtain the SF samples.

### Metabolic profiling

Extraction, detection and quantitative analysis of metabolites in samples were performed by Wuhan Metware Biotechnology Co., Ltd. (www.metware.cn). Briefly, hydrophilic and hydrophobic substances were extracted, and quality control (QC) samples were prepared by mixing sample extracts. Untargeted metabolic profiling was performed to detect metabolites in QC samples using ultra-performance liquid chromatography (UPLC; ExionLC AD; AB SCIEX; https://sciex.com.cn/) and quadrupole-time-of-flight (TripleTOF 6600; AB SCIEX). A new, specific, self-built database was then established based on the public MetWare database, and multi-ion pairs and retention times of metabolites identified by untargeted metabolic profiling. Widely targeted metabolic profiling in multiple reaction monitoring (MRM) mode was then performed using UPLC (ExionLC AD; https://sciex.com.cn/) and tandem mass spectrometry (QTRAP; https://sciex.com.cn/) to establish the metabolic profile of SF samples. QC samples were added to every 10 samples during the analysis, and precise quantification of metabolites was achieved based on the new database. The datasets presented in the study can be found in Metabolights database (accession number: MTBLS9662 and MTBLS9665) (17).

### Differential metabolites and metabolic pathways

Raw data from MRM metabolic profiling were subjected to further analysis using R software (18). Dispersion of samples was roughly visualized by principal component analysis (PCA). For different TMJOA groups (multiple categorical variables), orthogonal partial least squares discriminant analysis (OPLS-DA) was applied to remove irrelevant variables, and the variable importance in the projection (VIP) score of each metabolite was calculated to evaluate their contribution to the model. The reliability of the OPLS-DA model was evaluated by Q2 (predictability of the model) and R2Y (interpretability of the model for the categorical variable Y). One-way analysis of variance (ANOVA) with Tukey’s *post hoc* test was subsequently performed. Metabolites with VIP score >1 and P value <0.05 were considered differential metabolites. Differential metabolites with fold change for Mild versus Moderate groups and Moderate versus Severe groups both <1 or >1 were considered gradually increased or decreased metabolites. For different pain groups, unpaired Student’s t-test (two-tailed) was performed, and metabolites with P value <0.05 were considered differential metabolites. The metabolic pathways of differential metabolites were identified through Kyoto Encyclopedia of Genes and Genomes (KEGG) enrichment analysis.

### Weighted gene co-expression network analysis

WGCNA identified differentially co-expressed metabolite modules. Associations between modules and clinical information (mainly continuous variables) were calculated. Pearson correlation coefficients were employed to assess correlations between metabolites and MIO, and metabolites in modules were further analyzed by KEGG enrichment analysis.

### Machine learning for participant classification

Gradually increased or decreased metabolites among Mild, Moderate and Severe groups were utilized in machine learning. For further analysis, Moderate and Severe groups were combined, and the combined group was compared with the Mild group. Metabolites were screened by LASSO and random forest algorithms, and overlapping results were selected as the final biomarker panel. In the two groups, 2/3 samples were randomly and respectively selected as the training set, the remaining samples were selected as the validation set, and the prediction model was established by logistical regression. The predictive power of models was evaluated by the receiver operating characteristic (ROC) curve and area under the curve (AUC) was calculated.

## Results

### Patient characteristics

A total of 90 SF samples from patients with ADD-related TMJOA were collected during disc repositioning surgery for further metabolic profiling (**Figure 1A**). Patient data are listed in **Supplementary Table 1**. Based on TMJ MRI, patients were divided into three groups, Mild, Moderate and Severe, with 30 samples in each group. The age of patients in the three groups was 17.7 ± 2.7, 16.7 ± 1.7 and 17.8 ± 3.4 years, respectively. The proportion of females in the three groups was 80.0%, 96.7% and 76.7%, respectively. There were no significant differences in age (**Figure 1B**) and gender (**Figure 1C**) among the three groups. The MIO was 38.6 ± 12.0, 37.0 ± 7.5 and 36.8 ± 7.6 mm, respectively, and there were no differences among the groups (**Figure 1D**). The proportion of patients with pain was 34.5%, 66.7% and 61.5%, respectively, with a much higher proportion of patients with pain in the Moderate and Severe groups (**Figure 1E**). More than 80% of patients in the three groups exhibited joint clicking, and there was no significant difference among groups (**Figure 1F**).

### Overview of metabolic profiling

The total ion current (TIC) diagrams for the 10 QC samples overlapped significantly, and the intensity and retention time were consistent for both hydrophilic (**Supplementary Figure 1A**) and hydrophobic (**Supplementary Figure 1B**) metabolites. In qualitative and quantitative analyses of metabolites, MRM assessment of multimodal maps showed the detected metabolites, with each color-coded peak representing one metabolite (**Supplementary Figures 1C, D**). Pearson correlation analysis revealed high correlations among the 10 QC samples (r >0.99; **Supplementary Figure 1E**), indicating a stable detection process and high data reliability, and 1498 metabolites were eventually detected in samples. The top three types of metabolites were amino acids and their metabolites, benzene and substituted derivatives, and glycerophospholipids (**Figure 1G**).

### Differential metabolites in patients with different TMJOA grades

**Figure 2:**
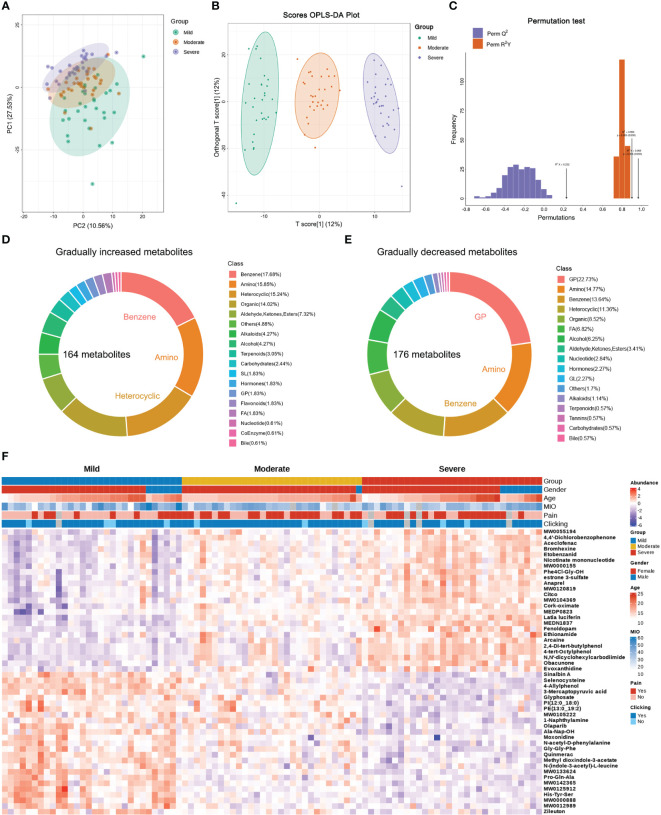
Differential metabolites in patients with different TMJOA grades. **(A)** Principal component analysis of SF samples. **(B)** Orthogonal partial least squares-discriminant analysis (OPLS-DA) of samples. **(C)** Subsequent permutation testing of the OPLS-DA model. **(D)** Overview of gradually increasing metabolites. **(E)** Overview of gradually decreasing metabolites. **(F)** Heatmap of the top 25 gradually decreasing and increasing metabolites.

Raw data were subjected to principal component analysis (PCA; **Figure 2A**) and orthogonal partial least squares-discriminant analysis (OPLS-DA; **Figure 2B**). Subsequent permutation tests confirmed the reliability of the OPLS-DA model with Q2 >0.9 and R2Y >0.9 (**Figure 2C**). There were differences between Mild, Moderate and Severe groups in both PCA and OPLS-DA maps. Based on fold change and VIP values, 164 gradually increasing metabolites (**Supplementary Table 2**) and 176 gradually decreasing metabolites (**Supplementary Table 3**) were screened out. The top three types of gradually increasing metabolites were benzene and substituted derivatives, amino acids and their metabolites, and heterocyclic compounds (**Figure 2D**). The top three types of gradually decreasing metabolites were glycerophospholipids, amino acids and their metabolites, and benzene and substituted derivatives (**Figure 2E**). The top 25 gradually decreasing or increasing metabolites are displayed in a heatmap (**Figure 2F**).

### Differences in metabolic pathways in patients with different TMJOA grades

**Figure 3:**
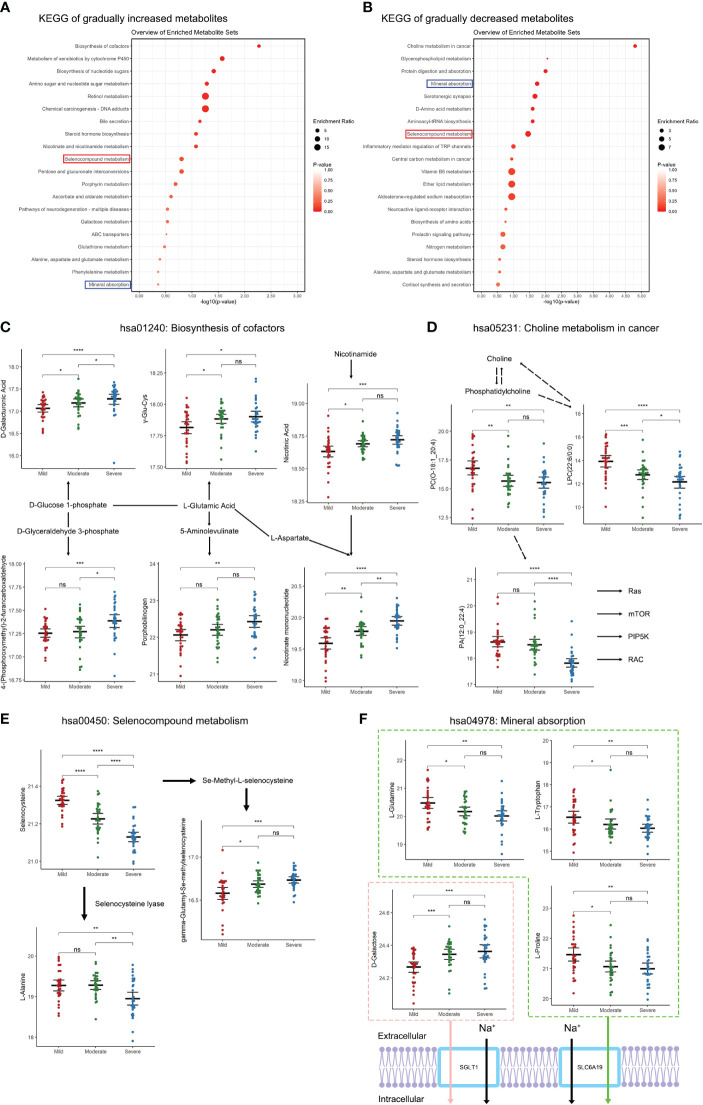
Changes in metabolic pathways of SF in patients with different TMJOA grades. **(A)** KEGG analysis of gradually increasing metabolites. **(B)** KEGG analysis of gradually decreasing metabolites. **(C)** Metabolites enriched in the biosynthesis of cofactors pathway. **(D)** Metabolites enriched in the choline metabolism in cancer pathway. **(E)** Metabolites enriched in the selenocompound metabolism pathway. **(F)** Metabolites enriched in the mineral absorption pathway. *, P < 0.05; **, P < 0.01; ***, P < 0.001; ****, P < 0.0001; ns, not significant.

To further analyze the functions of the differential metabolites, KEGG analysis was performed. Gradually increasing metabolites were mainly enriched in biosynthesis of cofactors, metabolism of xenobiotics by cytochrome P450, and biosynthesis of nucleotide sugars (**Figure 3A**). Gradually decreasing metabolites were mainly enriched in choline metabolism in cancer, glycerophospholipid metabolism, and protein digestion and absorption (**Figure 3B**). Notably, both gradually increasing and gradually decreasing metabolites were enriched in mineral absorption and selenocompound metabolism. With aggravation of bone destruction there were increases in D-glucose-related, L-Glutamic-acid-related and nicotinamide-related metabolism (**Figure 3C**). The decreasing metabolites, including phosphatidylcholine, phosphatidate acid and lysophosphatidylcholine, indicated downregulation of choline metabolism with aggravation of TMJOA (**Figure 3D**). Moreover, selenocysteine and its downstream metabolite L-alanine showed downregulation with aggravation of TMJOA, while another downstream metabolite of selenocysteine, gamma-glutamyl-Se-methylselenocysteine, showed upregulation (**Figure 3E**). Three metabolites associated with SLC6A19-related Na+ transport, namely L-glutamine, L-tryptophan, and L-proline, all gradually decreased in the three groups. However, D-galactose, associated with SGLT1-related Na+ transport, showed upregulation with aggravation of TMJOA (**Figure 3F**).

### Differential metabolites in patients with pain

**Figure 4:**
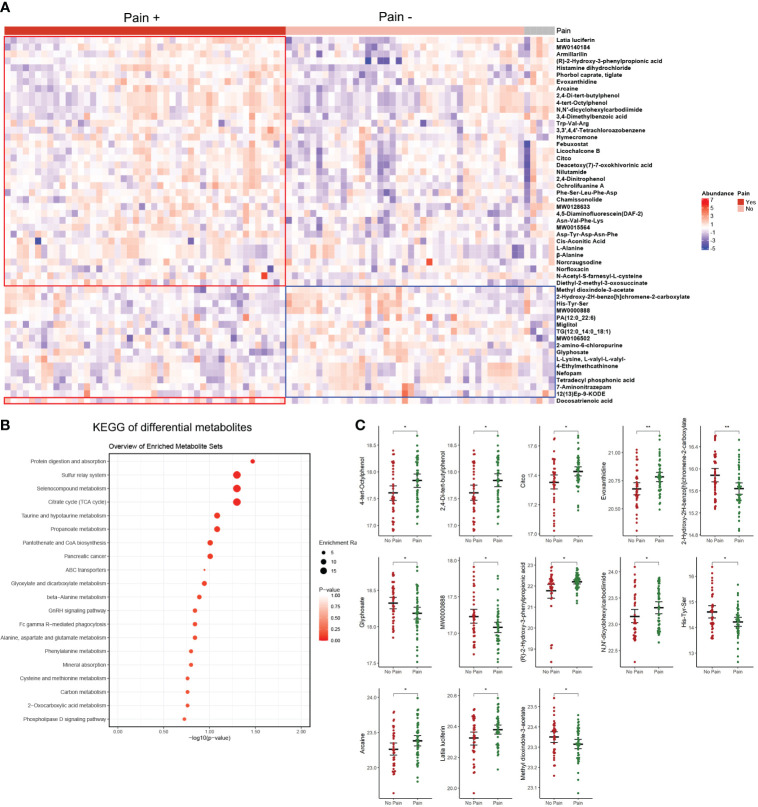
Differential analysis of patients with pain versus patients without pain. **(A)** Heatmap of differential metabolites. **(B)** KEGG analysis of differential metabolites. **(C)** The 13 metabolites overlapping with the top 50 differential metabolites detected in different TMJOA grades. *, P < 0.05; **, P < 0.01.

Pain is an important symptom in patients with TMJOA. Herein, 46 patients complained of pain while 39 patients reported no pain. Compared with patients without pain, differential analysis identified 37 upregulated metabolites and 16 downregulated metabolites in patients with pain (**Figure 4A**; **Supplementary Table 4**). The differential metabolites were mainly enriched in protein digestion and absorption, sulphur relay system, selenocompound metabolism, and tricarboxylic acid (TCA) or citrate cycle pathways (**Figure 4B**). Notably, 13 metabolites overlapped with the top 50 differential metabolites detected in different TMJOA grades (**Figure 4C**). These metabolites may be potential targets for treating bone destruction and pain in TMJOA.

### Associations between metabolites and MIO

**Figure 5:**
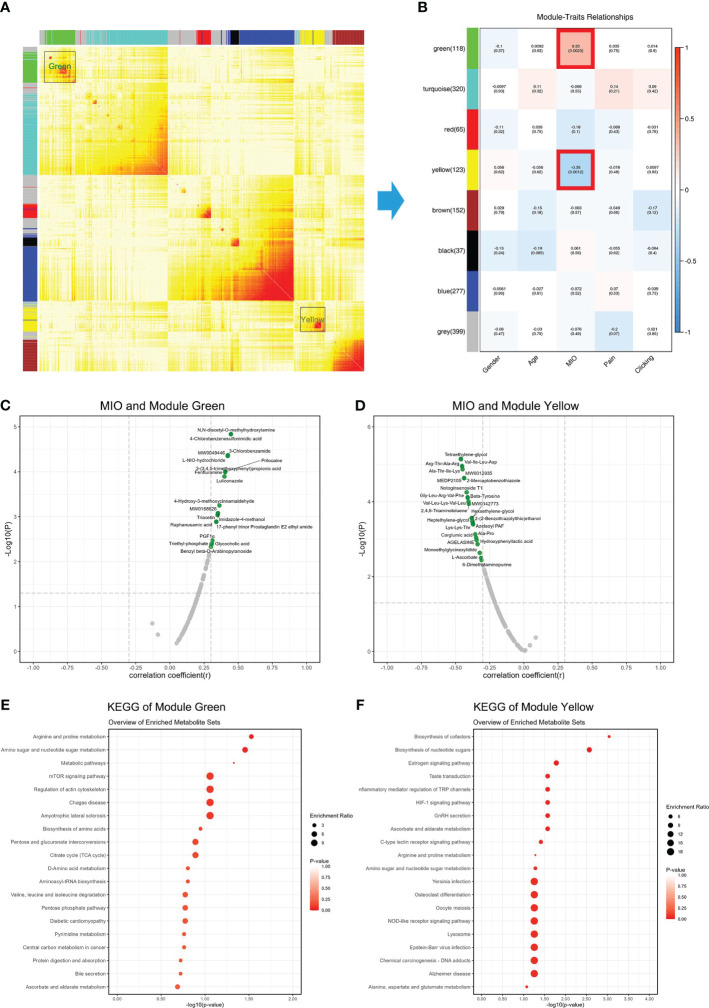
Associations between metabolites and MIO. **(A)** Weighted gene co-expression network analysis showing correlations among all detected metabolites. **(B)** Correlations between clinical information and gene modules. **(C)** Correlations between MIO and metabolites in the green module. **(D)** Correlations between MIO and metabolites in the yellow module. **(E)** KEGG analysis of metabolites in the green module. **(F)** KEGG analysis of metabolites in the yellow module.

WCGNA is used to cluster metabolites, and metabolites were divided into eight modules (**Figure 5A**). Correlations between modules and clinical information were examined, and metabolites in the green module (**Supplementary Table 5**) were positively correlated with MIO, while metabolites in the yellow module (**Supplementary Table 6**) were negatively correlated with MIO (**Figure 5B**). The 19 metabolites in the green module showed a stronger positive correlation with MIO (r >0.3; **Figure 5C**). The 26 metabolites in the yellow module showed a stronger negative correlation with MIO (r <-0.3; **Figure 5D**). Metabolites in the green module were enriched in arginine and proline metabolism, amino sugar and nucleotide sugar metabolism, and the mTOR signaling pathway (**Figure 5E**). Metabolites in the yellow module were enriched in biosynthesis of cofactors, biosynthesis of nucleotide sugars, and the estrogen signaling pathway (**Figure 5F**).

### Potential biomarkers for diagnosing moderate and severe TMJOA

**Figure 6:**
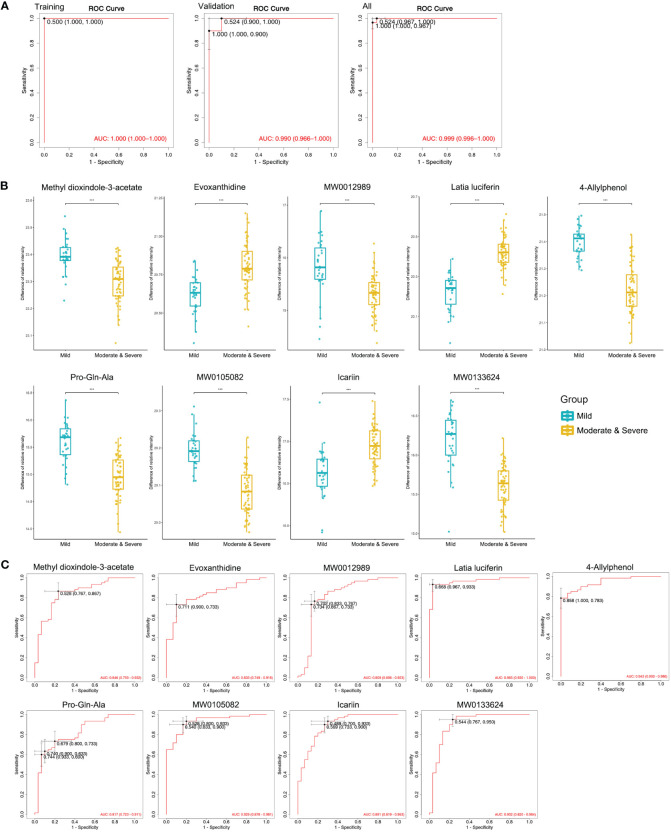
Machine learning to identify potential biomarkers for diagnosing moderate and severe TMJOA. **(A)** ROC curves of the models, including the selected biomarkers, established in the training set, the validation set, and all samples. **(B)** Abundance of metabolites in Mild and combined Moderate/Severe groups. **(C)** ROC curves of the selected biomarkers to diagnose Moderate and Severe TMJOA in all samples. ***, P < 0.001.

A decrease in condylar height is indicative of bone destruction. Thus, a combined Moderate and Severe group was compared with the Mild group to identify biomarkers with potential for diagnosing bone destruction in TMJOA. Nine metabolites were screened out (**Supplementary Table 7**) and logistical regression analysis confirmed the strong power of the diagnostic model, with AUC = 1.000 for the training set, AUC = 0.990 for the validation set, and AUC = 0.999 for all patients (**Figure 6A**). Only one sample was misdiagnosed in the validation set using the model (**Supplementary Figure 2A**). All nine metabolites showed significant difference between Mild and combined groups (**Figure 6B**), and the results were consistent for training and validation sets (**Supplementary Figure 2B**). Each metabolite displayed strong predictive power (AUC >0.8; **Figure 6C**) and the results were consistent for training (AUC >0.8; **Supplementary Figure 2C**) and validation (AUC >0.6) sets (**Supplementary Figure 2D**).

## Discussion

TMJOA is receiving increasing attention due to its negative influence on craniofacial growth, especially in adolescents and young adults (19, 20). In clinical work, the pathological changes and severity of TMJOA can be reflected by changes in the SF, synovial membrane, articular disc and condyle (16). Thus, these components are the main focus of investigations on the mechanism of OA (4). In the clinical work, changes of SF in TMJOA can often be observed by MRI, so the change of various metabolites in SF may directly contribute to TMJOA (21). Thus, the in-depth study of SF metabolites can contribute to explore the pathogenesis, diagnose and treatment of TMJOA.

Previous studies mainly focused on the metabolite changes in SF in knee OA. Consistent with our current work, these studies reported changes in lipid, sugar and amino acid derivatives in SF in knee OA (22–26). Zheng et al. (22) detected 81 metabolites in 25 knee OA patients and 10 controls, and found six metabolites were strongly associated with OA. Mickiewicz et al. (23) detected 141 metabolites in 68 human knee SF samples, and identified 11 metabolites as biomarkers of knee OA. Carlson et al. (25) detected 1233 metabolites in 13 samples, and reported 35 metabolites as potential biomarkers to diagnose OA. Jin et al. (26) reported the differences of lipid metabolites among the joints with infectious, degenerative, and traumatic diseases. However, in these studies, a relatively small number of metabolites or samples were detected and analyzed. For TMJ diseases, only one study reported the salivary metabolomics in females with temporomandibular disorders (27). Thus, there was a lack of studies on SF in human TMJOA.

The present study is the first study to investigate the changes of metabolites in SF in human TMJOA. Compared with the previous studies, a much larger number of human TMJ SF samples was assessed, and 1498 metabolites were identified using widely targeted metabolic profiling in the present study. The detected metabolites were mainly amino acids and their metabolites, benzene and substituted derivatives, and lipids. Nucleotide-related and carbohydrate-related metabolites accounted for only ~2% of detected metabolites. Notably, the types of benzene-related metabolites in SF of TMJOA were surprisingly large. The influence of benzene and substituted derivatives on immunity and inflammation has been explored in several studies (28, 29). However, few studies have investigated the role of benzene-related metabolites in OA, which needs further studies.

With increasing TMJOA grade, 164 gradually increasing and 176 gradually decreasing metabolites were detected. Among them, organics (especially benzene), amino acids and lipids were the most abundant metabolites. Notably, some hormones were included among these differential metabolites; oestrone and prostaglandin E2 were gradually increased in the three groups, while androstenedione and cortisol were gradually decreased. These results confirmed the important roles of hormones in TMJOA (30). Upon aggravation of TMJOA, biosynthesis of cofactors was increased. Consistently, previous studies also reported a close relationship between OA and glucose-related (31), glutamate-related (32), and nicotinamide-related (33) metabolism. Choline is an alpha7 nicotinic acetylcholine receptor agonist, and is related to pain in OA (34). In our study, no changes in choline-related metabolites were detected in patients with pain, but choline-related metabolism was decreased with increasing TMJOA grade, indicating a close relationship with TMJOA. Moreover, selenocompound metabolism and mineral absorption were altered in different TMJOA grades. Selenocompounds play an important role in osteoarthritis (35), which may be related to the regulation of reactive oxygen species (36), ferroptosis (37), and mitochondrial function (38). Thus, the changes in selenocompound found in TMJOA deserve further investigation. In addition, changes in pathways related to mineral absorption are also related to OA (39). For instance, Na^+^ transport is closely related to the inorganic phosphate absorption, and is associated with the progression of OA (40). These changes in metabolic pathways upon aggravation of bone destruction deserve further investigation.

In TMJOA patients with pain, 53 differential metabolites were screened out. Among them, 13 metabolites overlapped with the top 50 differential metabolites detected in different TMJOA grades, indicating a close relationship between pain and bone destruction in TMJOA. Further WGCNA analysis identified two modules of metabolites that were related to MIO. These may provide a basis for further analysis of the relationships between metabolites and symptoms.

Finally, a prediction model was established with strong predictive power. Nine metabolites are involved in the model, and may be considered potential biomarkers. Among them, methyl dioxindole-3-acetate, evoxanthidine and latia luciferin were differential metabolites in patients with different OA grades, and also differential metabolites in patients with pain. These three metabolites are heterocyclic compounds and aldehydes, and their roles in OA are poorly understood. Changes in these metabolites could reveal help to the mechanism of pain and bone destruction in TMJOA, and may provide potential therapeutic targets.

In summary, a metabolic profile was constructed and assessed using a much larger number of TMJOA patients than in previous studies, and the prediction model could contribute to clinical diagnosis of TMJOA grade. The results identify previously unknown metabolites in human SF of TMJOA, and provide an important basis for further research on the mechanism and treatment of TMJOA.

## Data availability statement

The datasets presented in this study can be found in online repositories. The names of the repository/repositories and accession number(s) can be found in the article/**Supplementary Material**.

## Ethics statement

The studies involving humans were approved by Human Research Ethics Committee of Shanghai Ninth People’s Hospital, Shanghai Jiao Tong University School of Medicine (approval no. SH9H-2020-T7-1). The studies were conducted in accordance with the local legislation and institutional requirements. Written informed consent for participation in this study was provided by the participants’ legal guardians/next of kin.

## Author contributions

DZ: Data curation, Formal analysis, Investigation, Software, Validation, Visualization, Writing – original draft. YZ: Data curation, Formal analysis, Funding acquisition, Software, Validation, Writing – original draft. SX: Data curation, Formal analysis, Investigation, Writing – original draft. PS: Conceptualization, Funding acquisition, Project administration, Resources, Supervision, Writing – review & editing. CY: Conceptualization, Funding acquisition, Project administration, Resources, Supervision, Writing – review & editing.
