# Supplementary material for: Metabolic profiling of synovial fluid in human temporomandibular joint osteoarthritis
Source: Front Immunol. 2024 Mar 11;15:1335181. doi: 10.3389/fimmu.2024.1335181 (PMC10961395; doi:10.3389/fimmu.2024.1335181)
Supplement: Supplementary file 1 [file DataSheet_1.docx]

Supplementary Material

# Supplementary Figures and Tables

## Supplementary Figures


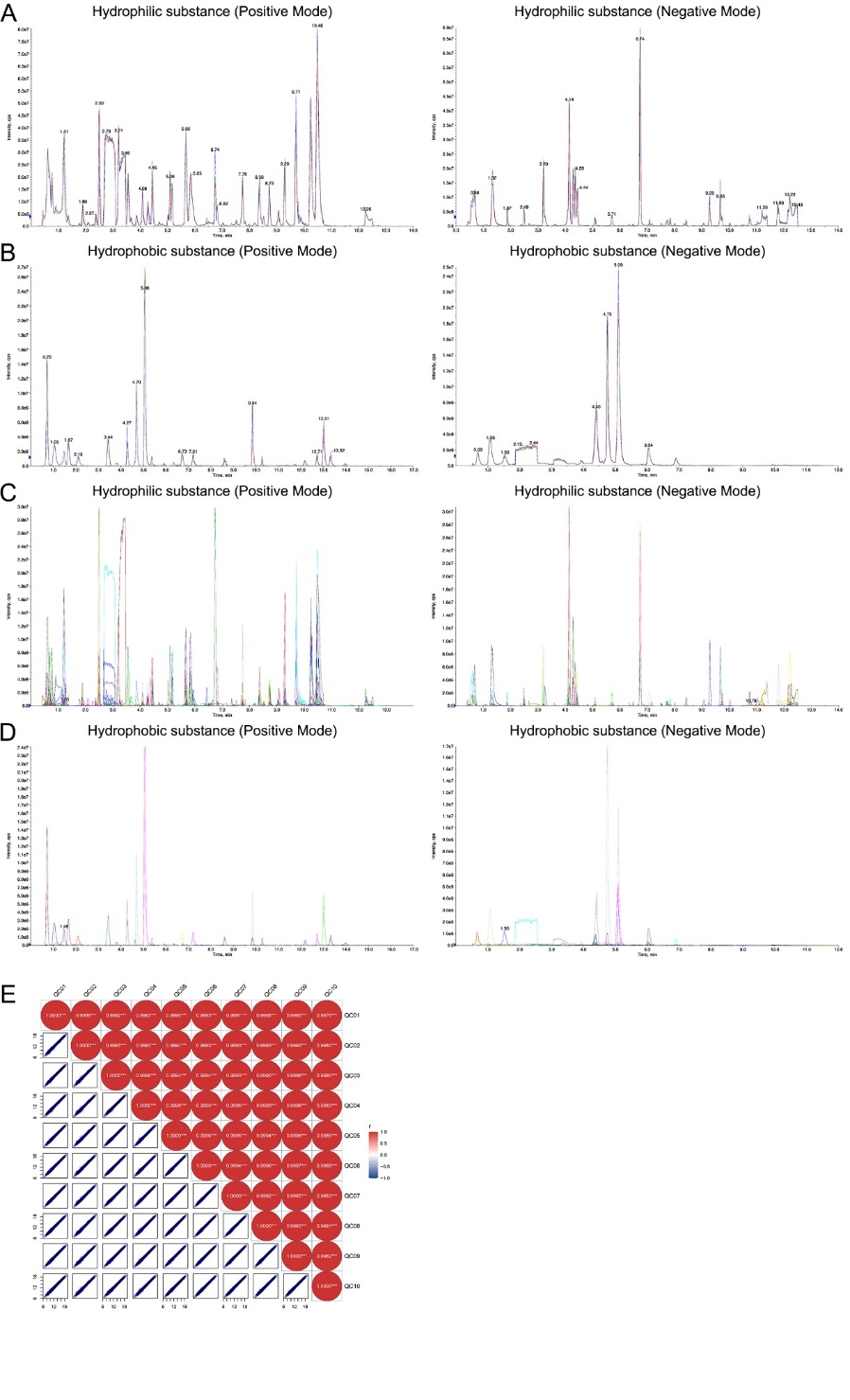


**Supplementary Figure 1.** Quality control (QC) of metabolic profiling. Total ion current overlapping diagrams of the 10 QC samples for hydrophilic (A) and hydrophobic (B) metabolites. Multimodal maps show the detected metabolites, with each color-coded peak representing one metabolite for hydrophilic (C) and hydrophobic (D) metabolites. (E) Pearson correlation analysis of the 10 QC samples.


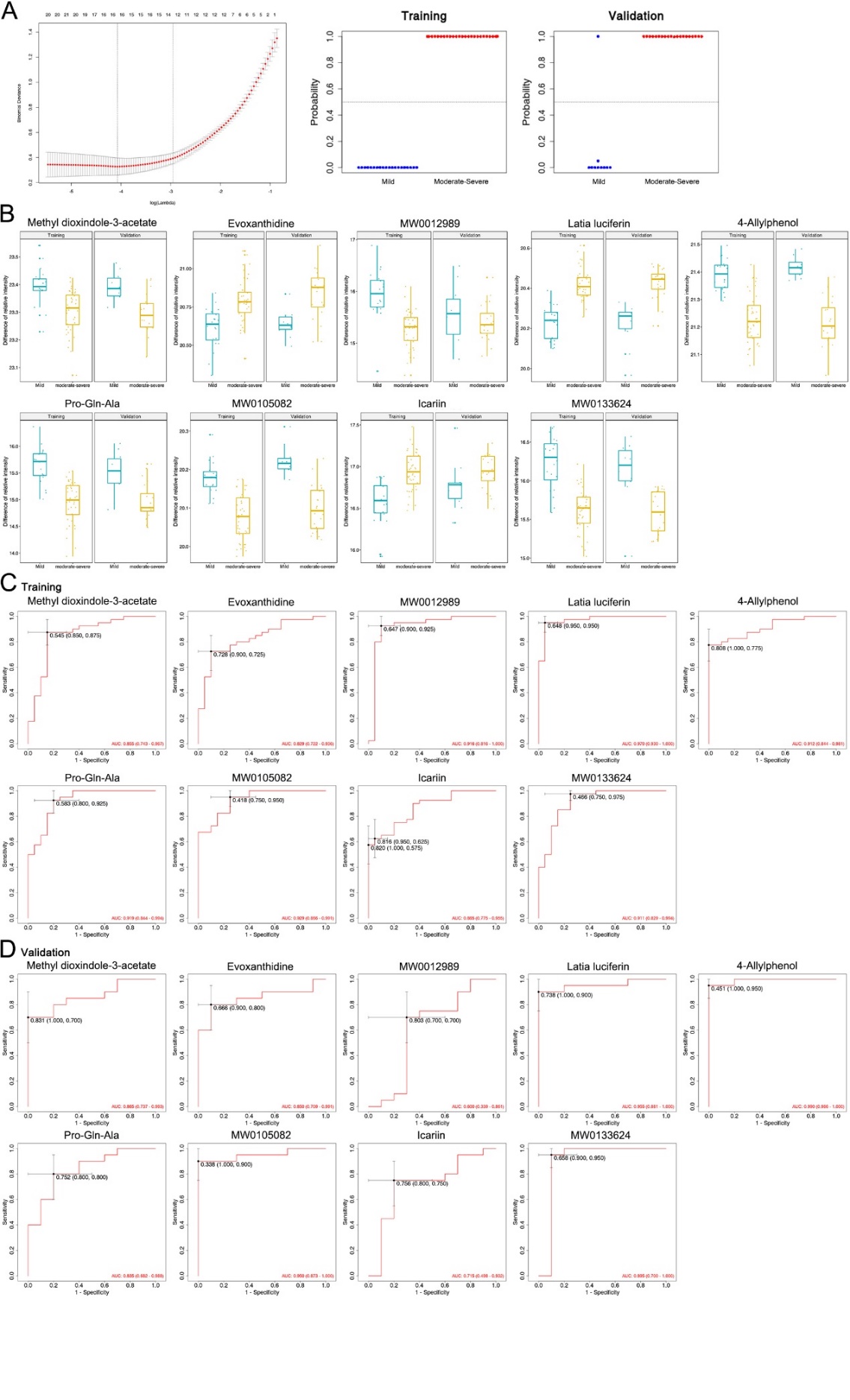


**Supplementary Figure 2.** Machine learning to identify potential biomarkers for diagnosing moderate and severe TMJOA. (A) Predictive power of the model for training and validation sets. (B) Abundance of the nine selected metabolites in training and validation sets. (C) ROC curves showing the predictive power of the nine metabolites in training and validation sets.

## Supplementary Tables

**Supplementary Table 1 Patient cohort**

| **Name** | **Group** | **Gender** | **Age** | **MIO, mm** | **Pain** | **Clicking** |
| --- | --- | --- | --- | --- | --- | --- |
| Mild_1 | Mild | Female | 17 | 21 | - | - |
| Mild_2 | Mild | Male | 17 | 50 | N | Y |
| Mild_3 | Mild | Female | 15 | 40 | N | Y |
| Mild_4 | Mild | Female | 14 | 38 | N | Y |
| Mild_5 | Mild | Female | 15 | 28 | N | N |
| Mild_6 | Mild | Female | 19 | 41 | N | Y |
| Mild_7 | Mild | Male | 19 | 40 | Y | N |
| Mild_8 | Mild | Female | 15 | 15 | Y | Y |
| Mild_9 | Mild | Female | 15 | 37 | N | Y |
| Mild_10 | Mild | Female | 18 | 32 | N | Y |
| Mild_11 | Mild | Female | 22 | 15 | N | Y |
| Mild_12 | Mild | Male | 20 | 43 | Y | Y |
| Mild_13 | Mild | Female | 19 | 20 | N | Y |
| Mild_14 | Mild | Female | 13 | 29 | N | Y |
| Mild_15 | Mild | Male | 17 | 36 | Y | Y |
| Mild_16 | Mild | Female | 16 | 42 | Y | Y |
| Mild_17 | Mild | Female | 22 | 44 | Y | Y |
| Mild_18 | Mild | Female | 12 | 52 | N | Y |
| Mild_19 | Mild | Female | 15 | 31 | Y | Y |
| Mild_20 | Mild | Female | 20 | 46 | Y | N |
| Mild_21 | Mild | Female | 19 | 46 | N | Y |
| Mild_22 | Mild | Female | 20 | 29 | N | Y |
| Mild_23 | Mild | Female | 19 | 54 | N | Y |
| Mild_24 | Mild | Female | 18 | 43 | N | Y |
| Mild_25 | Mild | Female | 19 | 43 | N | N |
| Mild_26 | Mild | Female | 18 | 30 | Y | Y |
| Mild_27 | Mild | Male | 15 | 60 | N | Y |
| Mild_28 | Mild | Female | 22 | 54 | N | Y |
| Mild_29 | Mild | Male | 21 | 60 | Y | Y |
| Mild_30 | Mild | Female | 20 | 39 | N | Y |
| Moderate_1 | Moderate | Female | 19 | - | Y | Y |
| Moderate_2 | Moderate | Female | 16 | 42 | Y | Y |
| Moderate_3 | Moderate | Female | 18 | 18 | N | Y |
| Moderate_4 | Moderate | Female | 19 | 30 | Y | Y |
| Moderate_5 | Moderate | Female | 17 | 35 | Y | Y |
| Moderate_6 | Moderate | Female | 18 | 30 | N | Y |
| Moderate_7 | Moderate | Female | 16 | 28 | Y | Y |
| Moderate_8 | Moderate | Female | 16 | 37 | N | Y |
| Moderate_9 | Moderate | Female | 17 | 35 | N | Y |
| Moderate_10 | Moderate | Female | 15 | 40 | Y | Y |
| Moderate_11 | Moderate | Female | 15 | 21 | Y | Y |
| Moderate_12 | Moderate | Female | 17 | - | Y | Y |
| Moderate_13 | Moderate | Female | 16 | 40 | N | Y |
| Moderate_14 | Moderate | Female | 19 | 37 | N | Y |
| Moderate_15 | Moderate | Female | 23 | 30 | Y | Y |
| Moderate_16 | Moderate | Female | 17 | 42 | Y | Y |
| Moderate_17 | Moderate | Female | 16 | 45 | N | Y |
| Moderate_18 | Moderate | Female | 17 | 37 | Y | N |
| Moderate_19 | Moderate | Female | 16 | 47 | Y | Y |
| Moderate_20 | Moderate | Female | 17 | 43 | Y | Y |
| Moderate_21 | Moderate | Female | 16 | 45 | Y | Y |
| Moderate_22 | Moderate | Male | 17 | 28 | Y | Y |
| Moderate_23 | Moderate | Female | 16 | 44 | N | Y |
| Moderate_24 | Moderate | Female | 17 | 45 | Y | Y |
| Moderate_25 | Moderate | Female | 15 | 40 | N | N |
| Moderate_26 | Moderate | Female | 15 | 40 | Y | Y |
| Moderate_27 | Moderate | Female | 15 | 35 | Y | Y |
| Moderate_28 | Moderate | Female | 16 | 35 | Y | Y |
| Moderate_29 | Moderate | Female | 15 | 48 | N | Y |
| Moderate_30 | Moderate | Female | 16 | 38 | Y | Y |
| Severe_1 | Severe | Female | 16 | 20 | - | - |
| Severe_2 | Severe | Female | 13 | 38 | - | - |
| Severe_3 | Severe | Female | 19 | 25 | Y | N |
| Severe_4 | Severe | Female | 17 | 37 | Y | Y |
| Severe_5 | Severe | Female | 23 | 30 | Y | Y |
| Severe_6 | Severe | Female | 14 | 48 | N | Y |
| Severe_7 | Severe | Female | 16 | 30 | Y | Y |
| Severe_8 | Severe | Female | 23 | 40 | Y | Y |
| Severe_9 | Severe | Female | 20 | 41 | Y | Y |
| Severe_10 | Severe | Female | 13 | 25 | N | Y |
| Severe_11 | Severe | Female | 21 | 28 | Y | Y |
| Severe_12 | Severe | Male | 20 | 45 | N | Y |
| Severe_13 | Severe | Male | 16 | 45 | - | - |
| Severe_14 | Severe | Female | 24 | 28 | Y | Y |
| Severe_15 | Severe | Female | 14 | 40 | Y | Y |
| Severe_16 | Severe | Male | 15 | 33 | Y | Y |
| Severe_17 | Severe | Female | 20 | 36 | Y | N |
| Severe_18 | Severe | Male | 23 | 51 | N | Y |
| Severe_19 | Severe | Female | 23 | 35 | Y | Y |
| Severe_20 | Severe | Female | 17 | 33 | N | Y |
| Severe_21 | Severe | Female | 17 | 32 | Y | Y |
| Severe_22 | Severe | Female | 16 | - | - | - |
| Severe_23 | Severe | Female | 14 | 32 | Y | Y |
| Severe_24 | Severe | Female | 12 | 45 | N | N |
| Severe_25 | Severe | Female | 15 | 40 | Y | Y |
| Severe_26 | Severe | Male | 17 | 45 | Y | Y |
| Severe_27 | Severe | Female | 19 | 40 | N | Y |
| Severe_28 | Severe | Female | 19 | 40 | N | Y |
| Severe_29 | Severe | Male | 21 | 44 | N | Y |
| Severe_30 | Severe | Male | 16 | 41 | N | Y |

MIO: Maximum inter-incisal opening.

Y: Yes.

N: No.

-: lost to follow-up.

**Supplementary Table 2 Gradually increasing metabolites in Mild, Moderate and Severe groups.**

| Index | Compounds | Class I | Class II |
| --- | --- | --- | --- |
| MW0152146 | Latia luciferin | Aldehyde,Ketones,Esters | Aldehydes |
| MEDN1837 | 2,6-Di-tert-butyl-4-(hydroxymethyl)phenol | Benzene and substituted derivatives | Benzene and substituted derivatives |
| MEDN1125 | N,N′-dicyclohexylcarbodiimide | Alcohol and amines | Polyamines |
| MEDN0747*042 | 2,4-Di-tert-butylphenol | Benzene and substituted derivatives | Phenolics |
| MEDN0731*042 | 4-tert-Octylphenol | Benzene and substituted derivatives | Phenolics |
| MW0052898 | Fenoldopam | Others | Medicine |
| MEDN0536 | estrone 3-sulfate | Hormones and hormone related compounds | Hormones and hormone related compounds |
| MW0005923 | Aceclofenac | Benzene and substituted derivatives | Benzene and substituted derivatives |
| MEDP2223 | Cork-oximate | Organic acid and Its derivatives | Organic acid and Its derivatives |
| MEDP0823 | (R)-2-Hydroxy-3-phenylpropionic acid | Organic acid and Its derivatives | Organic acid and Its derivatives |
| MW0168859 | Bromhexine | Benzene and substituted derivatives | Benzene and substituted derivatives |
| MW0000155 | (9R)-5-bromo-N-[(2S,4R,7S)-2-hydroxy-7-(2-methylpropyl)-5,8-dioxo-4-propan-2-yl-3-oxa-6,9-diazatricyclo[7.3.0.02,6]dodecan-4-yl]-7-methyl-6,6a,8,9-tetrahydro-4H-indolo[4,3-fg]quinoline-9-carboxamide | Alkaloids | Alkaloids |
| MW0004434 | 4,4'-Dichlorobenzophenone | Benzene and substituted derivatives | Benzene and substituted derivatives |
| MW0155423 | Phe4Cl-Gly-OH | Amino acid and Its metabolites | Small Peptide |
| MW0154673 | Obacunone | Terpenoids | Triterpene |
| MW0194041 | Anaprel | Alkaloids | Alkaloids |
| MW0007090 | Etobenzanid | Benzene and substituted derivatives | Benzene and substituted derivatives |
| MW0120819 | 5-(4-Acetoxybut-1-ynyl)-2,2'-bithiophene | Heterocyclic compounds | Heterocyclic compounds |
| MW0143727 | Citco | Benzene and substituted derivatives | Benzene and substituted derivatives |
| MW0103633 | Nicotinate mononucleotide | Nucleotide and Its metabolites | Nucleotide and Its metabolites |
| MW0110918 | Arcaine | Alcohol and amines | Polyamines |
| MW0055194 | N-(1,3-Dihydroxyoctadecan-2-YL)-6-[(7-nitro-2,1,3-benzoxadiazol-4-YL)amino]hexanamide | SL | Cer |
| MEDN1484 | Ethionamide | Heterocyclic compounds | Pteridines and derivatives |
| MW0104369 | 2-(S-Glutathionyl)acetyl glutathione | Amino acid and Its metabolites | Small Peptide |
| MW0149104 | Evoxanthidine | Heterocyclic compounds | Heterocyclic compounds |
| MW0128595 | [2,6-dihydroxy-4-(3,5,7-trihydroxy-3,4-dihydro-2H-1-benzopyran-2-yl)phenyl]oxidanesulfonic acid | Organic acid and Its derivatives | Organic acid and Its derivatives |
| MW0063337 | Retinyl ester | Aldehyde,Ketones,Esters | Esters |
| MEDP1814 | 2,5-Dimethyl-2,3-dihydrofuran-3-one | Heterocyclic compounds | Heterocyclic compounds |
| MEDN1261 | LPS(18:3) | GP | LPS |
| MW0063353 | Rishitinone | Alcohol and amines | Alcohols |
| MW0128773 | [4-(8-hydroxy-7-methoxy-4-oxo-3,4-dihydro-2H-1-benzopyran-2-yl)phenyl]oxidanesulfonic acid | Organic acid and Its derivatives | Organic acid and Its derivatives |
| MW0137941 | Dexibuprofen | Heterocyclic compounds | Heterocyclic compounds |
| MEDP2266 | Inositol 1,3,4-trisphosphate | Organic acid and Its derivatives | Phosphoric acids |
| MEDN1558 | L-Ascorbyl 6-palmitate | Aldehyde,Ketones,Esters | Esters |
| MEDN2298 | Elenaic acid | Organic acid and Its derivatives | Organic acid and Its derivatives |
| MW0000347 | Naltrindole | Heterocyclic compounds | Indole and Its derivatives |
| MEDN1722 | Piperic acid | Organic acid and Its derivatives | Organic acid and Its derivatives |
| MEDP1288 | Chrysophanol | Benzene and substituted derivatives | Benzene and substituted derivatives |
| MW0114715 | Leiocarposide | Benzene and substituted derivatives | Benzene and substituted derivatives |
| MW0124192 | Furmecyclox | Heterocyclic compounds | Heterocyclic compounds |
| MW0142603 | 3-(2'-Methylthio)ethylmalic acid | Organic acid and Its derivatives | Organic acid and Its derivatives |
| MW0169290 | Icariin | Flavonoids | Flavonoid |
| MW0006630 | Cloransulam-methyl | Benzene and substituted derivatives | Benzene and substituted derivatives |
| MW0115331 | Swertiamarin | Aldehyde,Ketones,Esters | Esters |
| MW0144209 | Abu-Nap-OH | Amino acid and Its metabolites | Amino acid derivatives |
| MW0155513 | Phorbol caprate, tiglate | Terpenoids | Ditepenoids |
| MW0003199 | 2-Methoxy-5-nitrophenol | Benzene and substituted derivatives | Benzene and substituted derivatives |
| MW0125809 | Nilutamide | Alcohol and amines | Amines |
| MW0133689 | 4'-Hydroxywarfarin | Heterocyclic compounds | Heterocyclic compounds |
| MEDN2050 | Daidzein | Benzene and substituted derivatives | Benzene and substituted derivatives |
| MEDP2047 | N-Acetyl-Asp-Glu | Amino acid and Its metabolites | Small Peptide |
| MW0139633 | Salvianolic acid A | Organic acid and Its derivatives | Organic acid and Its derivatives |
| MW0139182 | Ochratoxin A | Heterocyclic compounds | Heterocyclic compounds |
| MW0154804 | 2-(6-hydroxy-1,3-benzothiazol-2-yl)-1,3-thiazol-4(5H)-one | Benzene and substituted derivatives | Benzene and substituted derivatives |
| MW0054645 | Mactraxanthin | Aldehyde,Ketones,Esters | Esters |
| MW0104081 | Eugenol sulfate | Organic acid and Its derivatives | Sulfonic acids |
| MW0000382 | Ochrolifuanine A | Alkaloids | Alkaloids |
| MW0000009 | (1R,2R,9R)-1-piperidin-2-yl-3,15-diazatetracyclo[7.7.1.02,7.010,15]heptadecane | Alkaloids | Alkaloids |
| MEDN0678 | Ethylsalicylate | Organic acid and Its derivatives | Organic acid and Its derivatives |
| MEDN1966 | Pyrimidine-4-carboxylic acid | Heterocyclic compounds | Heterocyclic compounds |
| MW0002865 | 2-Chloro-4-biphenylol | Benzene and substituted derivatives | Benzene and substituted derivatives |
| MW0111319 | Thiolutin | Heterocyclic compounds | Heterocyclic compounds |
| MW0143282 | 4-Chlorochalcone | Aldehyde,Ketones,Esters | Ketones |
| ZINC14447816 | 7-Hydroxy-2,5-dimethyl-4H-1-benzopyran-4-one | Aldehyde,Ketones,Esters | Ketones |
| MW0006390 | Bisphenol AF | Benzene and substituted derivatives | Benzene and substituted derivatives |
| MW0126791 | Thioctic acid | Organic acid and Its derivatives | Organic acid and Its derivatives |
| MW0124101 | Fenazaflor | Others | Medicine |
| MEDL01749 | 4-Methylpentyl glucosinolate | Others | Glucosinolates |
| MW0157766 | Thr-Phe-Lys-Glu | Amino acid and Its metabolites | Small Peptide |
| MW0141395 | 14,15-Epoxyeicosa-5z-enoic acid | FA | Oxidized lipids |
| MW0146298 | Asp-Tyr-Asp-Asn-Phe | Amino acid and Its metabolites | Small Peptide |
| MW0152870 | Lys-Arg-Leu-Glu | Amino acid and Its metabolites | Small Peptide |
| MW0149438 | gamma-Glutamyl-Se-methylselenocysteine;5-L-Glutamyl-Se-methylselenocysteine | Amino acid and Its metabolites | Small Peptide |
| MW0000228 | D-Galactose | Carbohydrates and Its metabolites | Sugars |
| MW0143146 | 4-(3,4-Dichlorophenyl)-5-(4-pyridinyl)-2-thiazolamine | Heterocyclic compounds | Heterocyclic compounds |
| MW0126645 | Tenoxicam | Heterocyclic compounds | Heterocyclic compounds |
| MW0124123 | Flibanserin | Others | Medicine |
| MEDP1685 | Phytosphingosine | SL | SPH |
| MW0009783 | Sulfachlorpyridazine | Benzene and substituted derivatives | Benzene and substituted derivatives |
| MEDN1628 | 2,4-Quinolinediol | Heterocyclic compounds | Heterocyclic compounds |
| MW0015905 | Armillarilin | Benzene and substituted derivatives | Benzene and substituted derivatives |
| MW0000152 | (2S)-1-[(2E)-2-[(2S)-2,6-dicarboxy-2,3-dihydro-1H-pyridin-4-ylidene]ethylidene]-6-hydroxy-5-[(2S,3R,4S,5S,6R)-3,4,5-trihydroxy-6-(hydroxymethyl)tetrahydropyran-2-yl]oxy-indolin-1-ium-2-carboxylate | Alkaloids | Alkaloids |
| MW0104741 | 2-Ethylhexyl diphenyl phosphate | Aldehyde,Ketones,Esters | Esters |
| MW0138147 | (S)-2,3-Dihydro-5,7-dihydroxy-2-(4-hydroxyphenyl)-6,8-dimethyl-4-benzopyrone | Heterocyclic compounds | Heterocyclic compounds |
| MW0138306 | Gnaphaliin | Heterocyclic compounds | Heterocyclic compounds |
| MW0119987 | 4-(Methylnitrosamino)-1-(3-pyridyl-N-oxide)-1-butanol | Heterocyclic compounds | Heterocyclic compounds |
| MW0137864 | Dalbergin | Heterocyclic compounds | Heterocyclic compounds |
| MEDN0611 | 6β-hydroxytestosterone | Hormones and hormone related compounds | Hormones and hormone related compounds |
| MEDP2754 | 1-desoxymethylsphinganine | SL | SM |
| MW0002741 | 2-Benzyl-4-chlorophenol | Benzene and substituted derivatives | Benzene and substituted derivatives |
| MW0159118 | Val-Phe-Asp-Arg | Amino acid and Its metabolites | Small Peptide |
| MEDN1747 | 3,4-Dimethylbenzoic acid | Benzene and substituted derivatives | Benzene and substituted derivatives |
| MW0157590 | Thr-Asp-Lys-Arg | Amino acid and Its metabolites | Small Peptide |
| MEDN0098 | 2-Picolinic Acid | Heterocyclic compounds | Pteridines and derivatives |
| MEDN2302 | Licochalcone B | Benzene and substituted derivatives | Benzene and substituted derivatives |
| MW0000400 | Piperolactam A | Alkaloids | Alkaloids |
| MEDN1159 | FFA(15:1) | FA | FFA |
| MEDN1532 | D-Galacturonic Acid | Carbohydrates and Its metabolites | Sugar acids |
| MEDN1015 | Nicotinic Acid | CoEnzyme and vitamins | CoEnzyme and vitamins |
| MW0140438 | 4-(Phosphooxymethyl)-2-furancarboxaldehyde | Organic acid and Its derivatives | Organic acid and Its derivatives |
| MW0123498 | Deacetoxy(7)-7-oxokhivorinic acid | Organic acid and Its derivatives | Organic acid and Its derivatives |
| MW0013392 | 2-Aminohexadecanoic acid | Organic acid and Its derivatives | Organic acid and Its derivatives |
| MW0142592 | 3-HYDROXYDEOXODIHYDRODEOXYGEDUNIN | Others | Others |
| MEDN0622 | Porphobilinogen | Organic acid and Its derivatives | Organic acid and Its derivatives |
| MW0155355 | Phe-Thr-Lys-Lys | Amino acid and Its metabolites | Small Peptide |
| MW0110043 | Tris(2-carboxyethyl)phosphine hydrochloride | Organic acid and Its derivatives | Organic acid and Its derivatives |
| MW0062243 | Prostaglandin E2 p-benzamidophenyl ester | Hormones and hormone related compounds | Hormones and hormone related compounds |
| MEDN0235 | N-Acetyl-D-Glucosamine | Carbohydrates and Its metabolites | Sugar derivatives |
| MW0054376 | Linoleic Acid-d4 | FA | FFA |
| MW0004478 | 4,6-Dinitro-O-cresol | Benzene and substituted derivatives | Phenolics |
| MW0169320 | Isoginkgetin | Flavonoids | Flavonoid |
| MW0062120 | Pokeberrygenin | Terpenoids | Terpenoids |
| MEDN1059 | Tauroursodeoxycholic acid | Bile acids | Bile acids |
| MW0159341 | (3S)-versiconol acetate | Benzene and substituted derivatives | Benzene and substituted derivatives |
| MW0105002 | (2R)-3-(5-acetamido-2-hydroxyphenyl)sulfanyl-2-azaniumylpropanoate | Amino acid and Its metabolites | Amino acid derivatives |
| MW0106717 | Formothion | Aldehyde,Ketones,Esters | Aldehydes |
| MW0124310 | Hexobarbital | Heterocyclic compounds | Heterocyclic compounds |
| MW0006787 | Dichlorprop | Benzene and substituted derivatives | Benzene and substituted derivatives |
| MW0155333 | Phe-Ser-Leu-Phe-Asp | Amino acid and Its metabolites | Small Peptide |
| MW0063496 | Secologanin | Terpenoids | Terpenoids |
| MW0138808 | Lonchocarpol A | Heterocyclic compounds | Heterocyclic compounds |
| MEDN0429 | 3-Hydroxyhippuric Acid | Amino acid and Its metabolites | Amino acid derivatives |
| MW0108210 | Methazolamide | Alcohol and amines | Amines |
| MW0143804 | (2R,3S,5S,8R,9S,10S,13S,17R)-17-[(2S,3R,4R,5S)-3,4-dihydroxy-5,6-dimethylheptan-2-yl]-10,13-dimethyl-2,3,4,5,6,7,8,9,11,12,14,15,16,17-tetradecahydro-1H-cyclopenta[a]phenanthrene-2,3-diol | Aldehyde,Ketones,Esters | Ketones |
| MW0000175 | Cepharadione B | Alkaloids | Alkaloids |
| MW0168613 | 4-Methylumbelliferyl glucuronide | Heterocyclic compounds | Heterocyclic compounds |
| MW0155276 | Phe-Lys-Leu-Arg | Amino acid and Its metabolites | Small Peptide |
| MEDN0523 | Indole-3-lactic acid | Heterocyclic compounds | Indole and Its derivatives |
| MEDN2403 | LPG(17:0) | GP | LPG |
| MW0146457 | Avermectin A1a aglycone | Aldehyde,Ketones,Esters | Ketones |
| MW0149018 | Estradiol-3-sulfate | Organic acid and Its derivatives | Sulfonic acids |
| MEDP1067 | Palmitoyl-N-isopropylamide | Alcohol and amines | Amines |
| MW0144203 | Abu-His-OH | Amino acid and Its metabolites | Amino acid derivatives |
| MW0133854 | 5,12,14-trihydroxy-9-oxo-8,17-dioxatetracyclo[8.7.0.0(2),.0(1)(1),(1)]heptadeca-1(10),2(7),3,5,11(16),12,14-heptaen-13-yl acetate | Others | Others |
| MW0114953 | Glcnac6P | Organic acid and Its derivatives | Organic acid and Its derivatives |
| MW0053496 | Geranylgeraniol | Alcohol and amines | Alcohols |
| MW0155547 | Phytolaccagenic acid | Terpenoids | Triterpene |
| MW0007814 | Mibefradil | Benzene and substituted derivatives | Benzene and substituted derivatives |
| MW0007182 | Fluometuron | Benzene and substituted derivatives | Benzene and substituted derivatives |
| MW0145998 | Asn-TyrMe-OH | Amino acid and Its metabolites | Small Peptide |
| MW0109099 | Phe-Asp-Lys | Amino acid and Its metabolites | Small Peptide |
| MW0006633 | Clotrimazole | Benzene and substituted derivatives | Benzene and substituted derivatives |
| MW0106889 | alpha-Glutamylaspartic acid | Amino acid and Its metabolites | Small Peptide |
| MW0129857 | Mollicellin J | Others | Others |
| MEDN0074 | γ-Glu-Cys | Amino acid and Its metabolites | Small Peptide |
| MW0168825 | Bergapten | Heterocyclic compounds | Heterocyclic compounds |
| MEDN1606 | Acetanilide | Benzene and substituted derivatives | Benzene and substituted derivatives |
| MEDN2405 | LPG(19:0) | GP | LPG |
| MW0113946 | Asperuloside | Others | Others |
| MW0152618 | Leu-Nap-OH | Amino acid and Its metabolites | Small Peptide |
| MW0151226 | HoPhe-Tyr-OH | Aldehyde,Ketones,Esters | Esters |
| MW0157017 | Ser-Nap-OH | Amino acid and Its metabolites | Small Peptide |
| MW0109523 | Raltitrexed | Amino acid and Its metabolites | Amino acid derivatives |
| pmp000383 | Liquiritin | Flavonoids | Dihydroflavone |
| MW0152574 | Leu-Val-Val-Val-Gly | Amino acid and Its metabolites | Small Peptide |
| MW0154755 | O-phospho-D-serine | Organic acid and Its derivatives | Phosphoric acids |
| MW0170013 | Vulpinic acid | Benzene and substituted derivatives | Benzene and substituted derivatives |
| MW0118724 | 2-Methyl-3-(pyrimidin-2-YL)propanoic acid | Organic acid and Its derivatives | Organic acid and Its derivatives |
| MW0114405 | Erythritol | Carbohydrates and Its metabolites | Carbohydrates and Its metabolites |
| MW0168990 | CYCLOPIAZONIC ACID | Organic acid and Its derivatives | Sulfonic acids |
| MW0119481 | 3-Bromo-7-nitroindazole | Heterocyclic compounds | Heterocyclic compounds |
| MW0143827 | Obeticholic acid | Organic acid and Its derivatives | Organic acid and Its derivatives |
| MW0149874 | Glutaminyl-arginine | Amino acid and Its metabolites | Small Peptide |
| MW0109640 | Ser-Tyr-Arg | Amino acid and Its metabolites | Small Peptide |

**Supplementary Table 3 Gradually decreasing metabolites in Mild, Moderate and Severe groups**

| Index | Compounds | Class I | Class II |
| --- | --- | --- | --- |
| MW0103492 | 4-Allylphenol | Benzene and substituted derivatives | Phenolics |
| MW0105082 | 3-Mercaptopyruvic acid | Organic acid and Its derivatives | Organic acid and Its derivatives |
| MW0152780 | Selenocysteine | Amino acid and Its metabolites | Amino acid derivatives |
| MW0133624 | 2-(2-Amino-3-methoxyphenyl)-4H-1-benzopyran-4-one | Benzene and substituted derivatives | Benzene and substituted derivatives |
| MEDP2643 | Quinmerac | Benzene and substituted derivatives | Benzene and substituted derivatives |
| MW0142365 | 2-Hydroxy-2H-benzo[h]chromene-2-carboxylate | Organic acid and Its derivatives | Organic acid and Its derivatives |
| MW0126560 | Sinalbin A | Heterocyclic compounds | Heterocyclic compounds |
| MW0155826 | Pro-Gln-Ala | Amino acid and Its metabolites | Small Peptide |
| MW0151135 | His-Tyr-Ser | Amino acid and Its metabolites | Small Peptide |
| MEDP1670 | Gly-Gly-Phe | Amino acid and Its metabolites | Small Peptide |
| MW0144834 | Ala-Nap-OH | Amino acid and Its metabolites | Small Peptide |
| MW0000888 | [1-(2H-1,3-benzodioxol-5-yl)-3-(4-methoxy-1-benzofuran-5-yl)-3-oxopropoxy]sulfonic acid | Benzene and substituted derivatives | Benzene and substituted derivatives |
| MW0125912 | N-phenyl-4-(quinolin-2-ylmethyl)piperazine-1-carboxamide | Heterocyclic compounds | Heterocyclic compounds |
| MEDL01878 | Methyl dioxindole-3-acetate | Heterocyclic compounds | Indole and Its derivatives |
| MW0125967 | Olaparib | Heterocyclic compounds | Heterocyclic compounds |
| LIPID-N-1374 | PI(12:0_18:0) | GP | PI |
| MW0105222 | 4-Hydroxy-3-(sulfooxy)benzoic acid | Organic acid and Its derivatives | Organic acid and Its derivatives |
| MW0125223 | Moxonidine | Heterocyclic compounds | Heterocyclic compounds |
| MEDP0798 | 1-Naphthylamine | Benzene and substituted derivatives | Benzene and substituted derivatives |
| MW0012989 | 1-Stearoyl-2-arachidonoyl-sn-glycero-3-phospho-(1'-myo-inositol) | GP | PI |
| MEDP2478 | N-acetyl-D-phenylalanine | Amino acid and Its metabolites | Amino acid derivatives |
| MW0107191 | Glyphosate | Amino acid and Its metabolites | Amino acid derivatives |
| LIPID-N-1058 | PE(13:0_19:2) | GP | PE |
| MW0160374 | N-(indole-3-acetyl)-L-leucine | Organic acid and Its derivatives | Organic acid and Its derivatives |
| MW0127003 | Zileuton | Heterocyclic compounds | Heterocyclic compounds |
| MEDP2066 | Triethylenetetramine | Alcohol and amines | Polyamines |
| MEDL02687 | Feruloylcholine | Alkaloids | Alkaloids |
| MW0015784 | Androstenedione | Hormones and hormone related compounds | Hormones and hormone related compounds |
| MW0141079 | 1,2,3-Tris(chloromethoxy)propane | Others | Others |
| LIPID-N-1373 | PI(10:0_16:0) | GP | PI |
| LIPID-P-0392 | LPC(22:6/0:0) | GP | LPC |
| MW0125196 | MK 571(sodium salt) | Benzene and substituted derivatives | Benzene and substituted derivatives |
| MW0053495 | Geranylcitronellol | Alcohol and amines | Alcohols |
| MW0155411 | Phe-Val-Lys-Lys | Amino acid and Its metabolites | Small Peptide |
| MEDP1166 | PC(16:0/2:0) | GP | PC |
| MW0140318 | Indolmycenic acid | Heterocyclic compounds | Indole and Its derivatives |
| MEDP1653 | Glu-Glu | Amino acid and Its metabolites | Small Peptide |
| MW0106134 | cephalosporin C | Organic acid and Its derivatives | Organic acid and Its derivatives |
| MEDP1333 | LPC(0:0/20:4) | GP | LPC |
| MW0148315 | Desalkyl verapamil D617 | Heterocyclic compounds | Heterocyclic compounds |
| MW0108921 | N-tetradecanoyl-L-Homoserine Lactone | Aldehyde,Ketones,Esters | Esters |
| LIPID-P-0424 | LPE(18:2/0:0) | GP | LPE |
| MW0140542 | Geranyllinalool | Alcohol and amines | Alcohols |
| MW0123400 | 1-[(2-Chloro-1,3-thiazol-5-yl)methyl]-2-methyl-3-nitroguanidine | Heterocyclic compounds | Pyridine and pyridine derivatives |
| LIPID-N-1371 | PI(10:0_12:0) | GP | PI |
| LIPID-P-0384 | LPC(20:3/0:0) | GP | LPC |
| MW0054875 | Methyl caprylate | FA | Others |
| MEDP2118 | 2,3-Bis(4-hydroxyphenyl)propionitrile | Benzene and substituted derivatives | Benzene and substituted derivatives |
| MW0002184 | 2,4,5-Trichlorophenol | Benzene and substituted derivatives | Phenolics |
| LIPID-P-0379 | LPC(18:2/0:0) | GP | LPC |
| MW0015111 | 7-(6-Oct-1-enyl-2,3-diazabicyclo[2.2.1]hept-2-en-5-yl)hept-5-enoic acid | Others | Others |
| MW0108758 | Nelfinavir | Heterocyclic compounds | Heterocyclic compounds |
| MEDP2697 | 2-Thio-PAF | FA | Others |
| MEDP1701 | LPC(0:0/18:1) | GP | LPC |
| MEDP2728 | 3,4-Dichloroaniline | Benzene and substituted derivatives | Benzene and substituted derivatives |
| LIPID-N-1479 | PA(12:0_22:4) | GP | PA |
| LIPID-P-0383 | LPC(18:3/0:0) | GP | LPC |
| MW0145572 | Arg-Val-Ser | Amino acid and Its metabolites | Small Peptide |
| MW0013350 | 27-Norcholestanehexol | Alcohol and amines | Alcohols |
| MW0063695 | Daminozide | FA | Others |
| MEDP0891 | L-Tryptophanamide | Amino acid and Its metabolites | Amino acid derivatives |
| MEDP0373 | N-Acetylglycine | Amino acid and Its metabolites | Amino acid derivatives |
| MW0015566 | 3-[4-Methyl-1-(2-methylpropanoyl)-3-oxocyclohexyl]butanoic acid | Organic acid and Its derivatives | Organic acid and Its derivatives |
| MEDP0889 | Cortisol | Hormones and hormone related compounds | Hormones and hormone related compounds |
| MEDP2731 | 7alpha-Hydroxy-3-oxo-4-cholestenoic acid | Bile acids | Bile acids |
| MEDP0011 | L-Lysine | Amino acid and Its metabolites | Amino acids |
| MEDN0128 | 4-Hydroxy-3-methoxybenzaldehyde | Benzene and substituted derivatives | Phenolics |
| MW0114813 | Meglumine | Alcohol and amines | Amines |
| LIPID-P-0378 | LPC(0:0/18:2) | GP | LPC |
| MW0007593 | Mescaline | Benzene and substituted derivatives | Benzene and substituted derivatives |
| MW0140724 | (Z)-2-tetracos-15-enamidoethanesulfonic acid | Others | Others |
| MW0054562 | 1-(1Z-hexadecenyl)-sn-glycero-3-phosphocholine | GP | LPC-P |
| MW0008696 | N-(2,2,2-Trifluoroethyl)-N-{4-[2,2,2-trifluoro-1-hydroxy-1-(trifluoromethyl)ethyl]phenyl}benzenesulfonamide | Benzene and substituted derivatives | Benzene and substituted derivatives |
| LIPID-P-0031 | Carnitine C8:1 | FA | CAR |
| MEDP2792 | PC(O-1:0/O-16:0) | GP | PC-O |
| MW0010001 | Troxipide | Benzene and substituted derivatives | Phenolics |
| MEDP2664 | 4-Hydroxyquinoline | Benzene and substituted derivatives | Benzene and substituted derivatives |
| MW0007122 | Fenofibrate | Benzene and substituted derivatives | Benzene and substituted derivatives |
| LIPID-P-0423 | LPE(0:0/18:2) | GP | LPE |
| MW0139412 | Proanthocyanidin A2 | Tannins | Proanthocyanidins |
| MEDP0054 | L-Glutamine | Amino acid and Its metabolites | Amino acids |
| MW0052901 | Desferal-iron(III) | Heterocyclic compounds | Heterocyclic compounds |
| MW0054535 | 1-(9Z-octadecenoyl)-sn-glycero-3-phosphocholine | GP | LPC |
| MW0112781 | Dibenz(b,f)(1,4)oxazepine-10(11H)-carboxylic acid, 8-chloro-, 2-(1-oxo-3-(4-pyridinyl)propyl)hydrazide, monohydrochloride | Benzene and substituted derivatives | Benzene and substituted derivatives |
| MEDP2132*108 | 1-Aminocyclobutanecarboxylic acid | Amino acid and Its metabolites | Amino acid derivatives |
| MEDP0022*108 | L-Proline | Amino acid and Its metabolites | Amino acids |
| MEDP1272 | 20,26-dihydroxyecdysone | Hormones and hormone related compounds | Hormones and hormone related compounds |
| MEDP2326 | Cyclocreatine | Nucleotide and Its metabolites | Nucleotide and Its metabolites |
| MEDP1237 | Coniferol | Alcohol and amines | Alcohols |
| MEDP2104 | 3-Aminoquinoline | Benzene and substituted derivatives | Benzene and substituted derivatives |
| MEDP2349 | Ammeline | Heterocyclic compounds | Heterocyclic compounds |
| MW0006489 | Carbaryl | Benzene and substituted derivatives | Benzene and substituted derivatives |
| MEDN1908 | 5-Carboxyvanillic Acid | Organic acid and Its derivatives | Organic acid and Its derivatives |
| MW0144524 | Ala-Cys-Gln | Amino acid and Its metabolites | Small Peptide |
| LIPID-P-0399 | LPC(O-16:1) | GP | LPC-O |
| MW0115732 | Mefloquine hydrochloride | Heterocyclic compounds | Heterocyclic compounds |
| MW0006472 | Cannabidiolic acid | Benzene and substituted derivatives | Benzene and substituted derivatives |
| MEDP1127 | 1-acetylindole | Heterocyclic compounds | Indole and Its derivatives |
| MEDN2265 | Norethindrone acetate | Hormones and hormone related compounds | Hormones and hormone related compounds |
| MEDN0656 | Indoleacetaldehyde | Heterocyclic compounds | Indole and Its derivatives |
| MW0012538 | 16-Acetylpriverogenin A | Aldehyde,Ketones,Esters | Esters |
| MW0152160 | (R)-3-Amino-3-phenylpropanoic acid | Aldehyde,Ketones,Esters | Esters |
| MW0014397 | 4alpha,5alpha-Epoxy-11-eudesmen-3a-ol | Alcohol and amines | Alcohols |
| MEDP1742 | Ala-Phe | Amino acid and Its metabolites | Small Peptide |
| MW0110908 | 2-((3-Aminopropyl)amino)ethanethiol | Alcohol and amines | Alcohols |
| MW0054522 | 1-O-Hexadecyl-lyso-sn-glycero-3-phosphocholine | GP | LPC |
| ZINC153731 | Methyl 4-hydroxycinnamate | Aldehyde,Ketones,Esters | Esters |
| LIPID-N-1335 | PS(18:1_22:4) | GP | PS |
| MEDP2738 | 2-Hexadecanoylthio-1-ethylphosphorylcholine | FA | Others |
| MEDL00917 | LPE(0:0/20:4) | GP | LPE |
| MW0110487 | Zoledronic acid | Organic acid and Its derivatives | Organic acid and Its derivatives |
| MEDP0013*102 | L-Alanine | Amino acid and Its metabolites | Amino acids |
| MEDP1005*102 | β-Alanine | Amino acid and Its metabolites | Amino acids |
| LIPID-P-0368 | LPC(16:1/0:0) | GP | LPC |
| LIPID-P-0007 | Carnitine C6:0 | FA | CAR |
| MW0122332 | Dihydroneopterin phosphate | Organic acid and Its derivatives | Organic acid and Its derivatives |
| MW0110381 | L-Lysine, L-valyl-L-valyl- | Amino acid and Its metabolites | Small Peptide |
| LIPID-P-0354 | LPC(16:0/0:0) | GP | LPC |
| LIPID-P-0037 | Carnitine C14:1 | FA | CAR |
| LIPID-P-0352 | LPC(15:0/0:0) | GP | LPC |
| LIPID-P-0012 | Carnitine C9:0 | FA | CAR |
| LIPID-N-0478 | PI(16:0_18:1) | GP | PI |
| MEDP2703 | 4-Hydroxy-4-(pyridin-2-yl)butan-2-one | Heterocyclic compounds | Pyridine and pyridine derivatives |
| MW0136745 | 6-Methylcoumarin | Heterocyclic compounds | Heterocyclic compounds |
| MW0108011 | Lys-Lys-Arg | Amino acid and Its metabolites | Small Peptide |
| LIPID-P-0351 | LPC(0:0/15:0) | GP | LPC |
| MW0156393 | (3R,4S,5R)-5-[(3R)-3-[(3R,3aS,5aR,5bR,7aS,11aS,11bR,13aR,13bS)-5a,5b,8,8,11a,13b-hexamethyl-1,2,3,3a,4,5,6,7,7a,9,10,11,11b,12,13,13a-hexadecahydrocyclopenta[a]chrysen-3-yl]butyl]oxolane-2,3,4-triol | Terpenoids | Terpenoids |
| MEDP0380 | 2-(Dimethylamino)Guanosine | Nucleotide and Its metabolites | Nucleotide and Its metabolites |
| MEDP1419 | Carnitine C10:0 | FA | CAR |
| LIPID-P-0560 | PC(O-18:1_20:4) | GP | PC-O |
| LIPID-P-0355 | LPC(0:0/17:0) | GP | LPC |
| MW0151187 | Hodgkinsine | Heterocyclic compounds | Heterocyclic compounds |
| MEDP1836 | Nootkatone | Aldehyde,Ketones,Esters | Ketones |
| LIPID-P-0356 | LPC(17:0/0:0) | GP | LPC |
| LIPID-P-2271 | TG(10:0_10:0_12:0) | GL | TG |
| MW0053990 | Pregn-4-ene-3,20-dione, 17-(acetyloxy)- | Aldehyde,Ketones,Esters | Esters |
| MW0141088 | 1,2-Bis(4-hydroxyphenyl)-2-propanol | Heterocyclic compounds | Heterocyclic compounds |
| LIPID-P-0511 | PC(18:0_22:4) | GP | PC |
| MW0009478 | 4-(5H-Dibenzo[a,d]cyclohepten-5-ylidene)-1-[4-(2H-tetrazol-5-yl)butyl]-piperidine | Benzene and substituted derivatives | Benzene and substituted derivatives |
| MW0146971 | Calcipotriol | Alcohol and amines | Alcohols |
| MEDN1648 | CMPentylF | Organic acid and Its derivatives | Organic acid and Its derivatives |
| MEDP1073*124 | 6-O-methylguanine | Nucleotide and Its metabolites | Nucleotide and Its metabolites |
| MW0148131 | Alanyllactate | Amino acid and Its metabolites | Small Peptide |
| MEDP0336 | LPC(0:0/14:0) | GP | LPC |
| MW0006786 | Dichlorprop-P | Benzene and substituted derivatives | Benzene and substituted derivatives |
| LIPID-P-0353 | LPC(0:0/16:0) | GP | LPC |
| LIPID-N-0275 | PC(18:0_20:4) | GP | PC |
| LIPID-P-0350 | LPC(14:0/0:0) | GP | LPC |
| MEDP1183 | N-Acetylcadaverine | Alcohol and amines | Polyamines |
| LIPID-P-0395 | LPC(O-18:0) | GP | LPC-O |
| LIPID-P-0559 | PC(O-16:1_20:4) | GP | PC-O |
| MEDP1954 | N6-(2-Hydroxyethyl)adenosine | Nucleotide and Its metabolites | Nucleotide and Its metabolites |
| MW0139589 | Risedronic acid | Organic acid and Its derivatives | Organic acid and Its derivatives |
| MW0149665 | Gln-Gln-Gln-Glu-Gln | Amino acid and Its metabolites | Small Peptide |
| MEDP2334 | (R)-(-)-1-Amino-2-propanol | Alcohol and amines | Amines |
| LIPID-N-0063 | TXB2 | FA | Eicosanoid |
| LIPID-P-2284 | TG(14:0_14:0_16:1) | GL | TG |
| LIPID-N-0056 | PGE2 | FA | Eicosanoid |
| MEDP2024 | Dehydroascorbic acid | Organic acid and Its derivatives | Organic acid and Its derivatives |
| MEDP1699 | LPC(0:0/16:1) | GP | LPC |
| MW0106502 | Benzyl n-[(2s)-4-methyl-1-[[(2r)-4-methyl-1-[[(2s)-4-methyl-1-oxopentan-2-yl]amino]-1-oxopentan-2-yl]amino]-1-oxopentan-2-yl]carbamate | Organic acid and Its derivatives | Organic acid and Its derivatives |
| MW0054336 | alpha-L-Fucp-(1->3)-[beta-D-Galp-(1->4)]-D-GlcpNAc | Carbohydrates and Its metabolites | Sugars |
| MEDP0025 | L-Tryptophan | Amino acid and Its metabolites | Amino acids |
| MW0006976 | Enilconazole | Benzene and substituted derivatives | Benzene and substituted derivatives |
| MW0140425 | (2S)-2-hydroxy-3,4-diketopentyl phosphate | Organic acid and Its derivatives | Organic acid and Its derivatives |
| LIPID-P-1929 | LPC(16:2) | GP | LPC |
| MW0000168 | Capsaicin | Alkaloids | Alkaloids |
| MW0115093 | p-Coumaryl alcohol 4-O-glucoside | Benzene and substituted derivatives | Benzene and substituted derivatives |
| MEDP2696 | Traumatin | Organic acid and Its derivatives | Organic acid and Its derivatives |
| MEDP0526 | 6-Dimethylaminopurine | Nucleotide and Its metabolites | Nucleotide and Its metabolites |
| LIPID-P-0771 | TG(10:0_16:0_12:0) | GL | TG |
| MW0000724 | Shogaol | Benzene and substituted derivatives | Phenolics |
| MW0123894 | Ethirimol | Heterocyclic compounds | Heterocyclic compounds |
| MW0146364 | Asp-Nap-OH | Amino acid and Its metabolites | Small Peptide |
| MEDP1901 | Carnitine C9:1-OH | FA | CAR |
| MEDP1316 | MG(0:0/16:0/0:0) | GL | MG |

**Supplementary Table 4 Differential metabolites in patients with pain versus patients without pain**

| Index | Compounds | Class I | Class II |
| --- | --- | --- | --- |
| LIPID-P-0829 | TG(12:0_14:0_18:1) | GL | TG |
| MW0149104 | Evoxanthidine | Heterocyclic compounds | Heterocyclic compounds |
| MW0000382 | Ochrolifuanine A | Alkaloids | Alkaloids |
| MW0142365 | 2-Hydroxy-2H-benzo[h]chromene-2-carboxylate | Organic acid and Its derivatives | Organic acid and Its derivatives |
| MEDP2439 | 2-amino-6-chloropurine | Nucleotide and Its metabolites | Nucleotide and Its metabolites |
| MW0155513 | Phorbol caprate, tiglate | Terpenoids | Ditepenoids |
| MW0015564 | [(1S,6S,7S,8R,9R,13R,14R,16S,18R)-8-acetyloxy-11-ethyl-5,7,14-trihydroxy-6,16,18-trimethoxy-13-(methoxymethyl)-11-azahexacyclo[7.7.2.12,5.01,10.03,8.013,17]nonadecan-4-yl] benzoate | Benzene and substituted derivatives | Benzene and substituted derivatives |
| MW0111056 | Histamine dihydrochloride | Amino acid and Its metabolites | Small Peptide |
| MEDN0203 | Cis-Aconitic Acid | Organic acid and Its derivatives | Organic acid and Its derivatives |
| MW0125809 | Nilutamide | Alcohol and amines | Amines |
| MW0143727 | Citco | Benzene and substituted derivatives | Benzene and substituted derivatives |
| MEDN0747*042 | 2,4-Di-tert-butylphenol | Benzene and substituted derivatives | Phenolics |
| MEDN0731*042 | 4-tert-Octylphenol | Benzene and substituted derivatives | Phenolics |
| MW0015905 | Armillarilin | Benzene and substituted derivatives | Benzene and substituted derivatives |
| MW0145959 | Asn-Val-Phe-Lys | Amino acid and Its metabolites | Small Peptide |
| MEDP2099 | 4,5-Diaminofluorescein(DAF-2) | Benzene and substituted derivatives | Benzene and substituted derivatives |
| MEDN1747 | 3,4-Dimethylbenzoic acid | Benzene and substituted derivatives | Benzene and substituted derivatives |
| MW0107191 | Glyphosate | Amino acid and Its metabolites | Amino acid derivatives |
| MW0125183 | Miglitol | Heterocyclic compounds | Heterocyclic compounds |
| MW0000888 | [1-(2H-1,3-benzodioxol-5-yl)-3-(4-methoxy-1-benzofuran-5-yl)-3-oxopropoxy]sulfonic acid | Benzene and substituted derivatives | Benzene and substituted derivatives |
| MW0160208 | Norcraugsodine | Benzene and substituted derivatives | Phenolics |
| MW0155333 | Phe-Ser-Leu-Phe-Asp | Amino acid and Its metabolites | Small Peptide |
| MW0128633 | [3-(2H-1,3-benzodioxol-5-yl)-1-(4-methoxy-1-benzofuran-5-yl)-3-oxopropoxy]sulfonic acid | Organic acid and Its derivatives | Organic acid and Its derivatives |
| LIPID-N-1499 | PA(12:0_22:6) | GP | PA |
| MW0002287 | 2,4-Dinitrophenol | Benzene and substituted derivatives | Phenolics |
| MW0113040 | 4-Ethylmethcathinone | Aldehyde,Ketones,Esters | Ketones |
| MW0015479 | 12(13)Ep-9-KODE | FA | Oxidized lipids |
| MW0138422 | Hymecromone | Others | Medicine |
| MW0119149 | 3,3',4,4'-Tetrachloroazobenzene | Benzene and substituted derivatives | Benzene and substituted derivatives |
| MW0017031 | Chamissonolide | Heterocyclic compounds | Heterocyclic compounds |
| MEDN2302 | Licochalcone B | Benzene and substituted derivatives | Benzene and substituted derivatives |
| MEDN1719 | Tetradecyl phosphonic acid | Organic acid and Its derivatives | Organic acid and Its derivatives |
| MW0111202 | Nefopam | Heterocyclic compounds | Heterocyclic compounds |
| MW0123498 | Deacetoxy(7)-7-oxokhivorinic acid | Organic acid and Its derivatives | Organic acid and Its derivatives |
| MW0158282 | Trp-Val-Arg | Amino acid and Its metabolites | Small Peptide |
| MEDP0013*102 | L-Alanine | Amino acid and Its metabolites | Amino acids |
| MEDP1005*102 | β-Alanine | Amino acid and Its metabolites | Amino acids |
| MW0146298 | Asp-Tyr-Asp-Asn-Phe | Amino acid and Its metabolites | Small Peptide |
| MW0106502 | Benzyl n-[(2s)-4-methyl-1-[[(2r)-4-methyl-1-[[(2s)-4-methyl-1-oxopentan-2-yl]amino]-1-oxopentan-2-yl]amino]-1-oxopentan-2-yl]carbamate | Organic acid and Its derivatives | Organic acid and Its derivatives |
| MW0152146 | Latia luciferin | Aldehyde,Ketones,Esters | Aldehydes |
| MEDP0823 | (R)-2-Hydroxy-3-phenylpropionic acid | Organic acid and Its derivatives | Organic acid and Its derivatives |
| MW0052460 | Docosatrienoic acid | FA | FFA |
| MW0169568 | Norfloxacin | Others | Medicine |
| MW0122354 | 7-Aminonitrazepam | Heterocyclic compounds | Heterocyclic compounds |
| MEDN1125 | N,N′-dicyclohexylcarbodiimide | Alcohol and amines | Polyamines |
| MW0169519 | N-Acetyl-S-farnesyl-L-cysteine | Amino acid and Its metabolites | Amino acid derivatives |
| MW0140184 | (1-((1-Methylpiperidin-2-yl)methyl)-1H-indol-3-yl)(naphthalen-1-yl)methanone | Aldehyde,Ketones,Esters | Ketones |
| MEDL01878 | Methyl dioxindole-3-acetate | Heterocyclic compounds | Indole and Its derivatives |
| MW0110918 | Arcaine | Alcohol and amines | Polyamines |
| MEDP1470 | Diethyl-2-methyl-3-oxosuccinate | Aldehyde,Ketones,Esters | Aldehydes |
| MW0151135 | His-Tyr-Ser | Amino acid and Its metabolites | Small Peptide |
| MW0110381 | L-Lysine, L-valyl-L-valyl- | Amino acid and Its metabolites | Small Peptide |
| MW0124099 | Febuxostat | Others | Medicine |

**Supplementary Table 5 Metabolites in the green module in WGCNA**

| Index | Compounds | Class I | Class II |
| --- | --- | --- | --- |
| MEDP2719 | (2E,4E)-6-(2-aminophenyl)-2-hydroxy-6-oxohexa-2,4-dienoic acid | Organic acid and Its derivatives | Organic acid and Its derivatives |
| MEDN2099 | (2s)-2-Amino-4-sulfinobutanoic acid | Amino acid and Its metabolites | Amino acid derivatives |
| MEDP2126 | (4-Ethoxyphenyl)urea | Benzene and substituted derivatives | Benzene and substituted derivatives |
| MEDP1101 | (R)-(-)-3-Hydroxybutyric-acid-methyl-ester | Aldehyde,Ketones,Esters | Esters |
| MEDN0689 | 2,5-Furandicarboxylicacid | Organic acid and Its derivatives | Organic acid and Its derivatives |
| MEDP2534 | Raphanusamic acid | Organic acid and Its derivatives | Organic acid and Its derivatives |
| MEDP2109 | 3-(3,4,5-trimethoxyphenyl)propionic acid | Organic acid and Its derivatives | Organic acid and Its derivatives |
| MEDP2663 | 3-Chlorobenzamide | Benzene and substituted derivatives | Benzene and substituted derivatives |
| MEDN0228*020 | D-Arabinose | Carbohydrates and Its metabolites | Sugars |
| MEDN0225*020 | D-Xylose | Carbohydrates and Its metabolites | Sugars |
| MEDP2322 | L-NIO-hydrochloride | Amino acid and Its metabolites | Amino acid derivatives |
| MEDP1318 | LPC(0:0/22:4) | GP | LPC |
| MEDP0010 | L-Arginine | Amino acid and Its metabolites | Amino acids |
| MEDN1931*020 | L-lyxose | Carbohydrates and Its metabolites | Carbohydrates and Its metabolites |
| MEDN2107 | Lys-Gly | Amino acid and Its metabolites | Small Peptide |
| MEDP1780 | Leu-Phe | Amino acid and Its metabolites | Small Peptide |
| MEDP0049 | L-Asparagine Anhydrous | Amino acid and Its metabolites | Amino acids |
| MEDP2499 | Asn-Asn | Amino acid and Its metabolites | Small Peptide |
| MEDN1616 | Val-Pro-Leu | Amino acid and Its metabolites | Small Peptide |
| MEDP1052 | N,N-diacetyl-O-methylhydroxylamine | Alcohol and amines | Polyamines |
| MEDN2201 | N-Phenylanthranilic acid | Benzene and substituted derivatives | Benzene and substituted derivatives |
| MEDP0729 | Barbituric acid | Heterocyclic compounds | Heterocyclic compounds |
| MEDP2312*134 | Gly-Lys | Amino acid and Its metabolites | Small Peptide |
| MEDP1741 | Agmatine | Alcohol and amines | Polyamines |
| MEDP1006 | Creatine | Organic acid and Its derivatives | Organic acid and Its derivatives |
| MEDP2318 | Lys-Ser | Amino acid and Its metabolites | Small Peptide |
| MEDP0535 | Triethyl-phosphate | Organic acid and Its derivatives | Phosphoric acids |
| MEDN2088 | Imidazole-4-methanol | Heterocyclic compounds | Heterocyclic compounds |
| MEDP1836 | Nootkatone | Aldehyde,Ketones,Esters | Ketones |
| MEDP1802 | Proline betaine | Amino acid and Its metabolites | Amino acid derivatives |
| MEDP0236 | D-Gluconic Acid | Carbohydrates and Its metabolites | Sugar acids |
| MEDP1809 | Triacetin | Aldehyde,Ketones,Esters | Esters |
| MEDN1224 | Bis(1-inositol)-3,1'-phosphate 1-phosphate | Alcohol and amines | Alcohols |
| MEDP1490 | Cis-4-Hydroxy-L-Proline | Amino acid and Its metabolites | Amino acid derivatives |
| MEDN0203 | Cis-Aconitic Acid | Organic acid and Its derivatives | Organic acid and Its derivatives |
| MW0049446 | [(2S)-3-hydroxy-2-octanoyloxypropyl] tridecanoate | GL | DG |
| MW0012083 | 11b-Hydroxyprogesterone | Aldehyde,Ketones,Esters | Ketones |
| MW0142513 | 2-O-alpha-mannosyl-D-glycerate | Organic acid and Its derivatives | Organic acid and Its derivatives |
| MW0119481 | 3-Bromo-7-nitroindazole | Heterocyclic compounds | Heterocyclic compounds |
| MW0142997 | 3-Hydroxyquinoline-2-carboxylic acid | Organic acid and Its derivatives | Organic acid and Its derivatives |
| MW0105422 | Acetylhomoserine | Amino acid and Its metabolites | Amino acid derivatives |
| MW0106889 | alpha-Glutamylaspartic acid | Amino acid and Its metabolites | Small Peptide |
| MW0113970 | Benzyl beta-D-Arabinopyranoside | Others | Others |
| MW0123123 | Bis(2-methyl-3-furyl) tetrasulfide | Heterocyclic compounds | Heterocyclic compounds |
| MEDL00401 | Confertifoline | Aldehyde,Ketones,Esters | Esters |
| MW0114546 | Glucosamine 1-phosphate | Organic acid and Its derivatives | Organic acid and Its derivatives |
| MW0053997 | Hydroxyvalerenic Acid | Organic acid and Its derivatives | Organic acid and Its derivatives |
| MW0151417 | Ile-Glu-His-Lys | Amino acid and Its metabolites | Small Peptide |
| MW0152140 | L-beta-aspartyl-L-arginine | Amino acid and Its metabolites | Amino acids |
| MW0152806 | Luliconazole | Benzene and substituted derivatives | Benzene and substituted derivatives |
| MW0153924 | Mirasan | Benzene and substituted derivatives | Benzene and substituted derivatives |
| MW0130384 | Mucronustyrene | Benzene and substituted derivatives | Benzene and substituted derivatives |
| MW0009671 | Rhein | Benzene and substituted derivatives | Benzene and substituted derivatives |
| MW0157352 | Tauropine | Amino acid and Its metabolites | Amino acids |
| MW0009887 | Tetrachlorosalicylanilide | Benzene and substituted derivatives | Phenolics |
| MW0141968 | Tetrahydrodipicolinate | Organic acid and Its derivatives | Organic acid and Its derivatives |
| MW0103070 | Valerenic acid | Organic acid and Its derivatives | Organic acid and Its derivatives |
| MW0170013 | Vulpinic acid | Benzene and substituted derivatives | Benzene and substituted derivatives |
| MW0000323 | (1R,2R,9S,10S)-7,15-diazatetracyclo[7.7.1.02,7.010,15]heptadecan-6-one | Alkaloids | Alkaloids |
| MW0000330 | (5beta,6beta,7beta,11alpha)-Matridin-15-one | Alkaloids | Alkaloids |
| MW0140731 | (Z)-(4-hydroxyphenyl)acetaldehyde oxime | Benzene and substituted derivatives | Phenolics |
| MW0137701 | (Z)-3,5,4'-Trimethoxystilbene | Heterocyclic compounds | Heterocyclic compounds |
| MW0168457 | 1-Hexadecyl-sn-glycerol 3-phosphate | GP | LPA |
| MW0012648 | 17-phenyl trinor Prostaglandin E2 ethyl amide | Hormones and hormone related compounds | Hormones and hormone related compounds |
| MW0109380 | 2-[(Pyrrolidinium-2-ylcarbonyl)amino]propanoate | Amino acid and Its metabolites | Small Peptide |
| MW0006042 | 2-Hydroxy-6-pentadecylbenzoic acid | Benzene and substituted derivatives | Benzene and substituted derivatives |
| MW0159850 | 2,3-Dihydroxy-4-phosphonooxybutanoic acid | Organic acid and Its derivatives | Organic acid and Its derivatives |
| MW0004852 | 4-Chlorobenzenesulfonimidic acid | Benzene and substituted derivatives | Benzene and substituted derivatives |
| MW0113040 | 4-Ethylmethcathinone | Aldehyde,Ketones,Esters | Ketones |
| MW0005059 | 4-Hydroxy-3-methoxycinnamaldehyde | Benzene and substituted derivatives | Phenolics |
| MW0168626 | 5(Z),8(Z),11(Z)-Eicosatrienoic Acid methyl ester | Aldehyde,Ketones,Esters | Esters |
| MW0105414 | Acetoacetic acid | Organic acid and Its derivatives | Organic acid and Its derivatives |
| MW0144735 | Ala-Ser-Ser-Thr-Lys | Amino acid and Its metabolites | Small Peptide |
| MW0122763 | Alfuzosin | Heterocyclic compounds | Heterocyclic compounds |
| MW0016129 | b-Kessyl ketone | Aldehyde,Ketones,Esters | Ketones |
| MW0006346 | Betaxolol | Benzene and substituted derivatives | Phenolics |
| MW0106068 | Bupivacaine | Benzene and substituted derivatives | Benzene and substituted derivatives |
| MW0055212 | CerP(d18:1/24:0) | SL | Cer |
| MW0106147 | Chlorthiophos | Organic acid and Its derivatives | Phosphoric acids |
| FDATP00473 | Clomipramine hydrochloride | Others | Medicine |
| MW0148310 | D-Erythrulose | Carbohydrates and Its metabolites | Sugars |
| MW0103526 | Deoxyuridine triphosphate | Nucleotide and Its metabolites | Nucleotide and Its metabolites |
| MW0148636 | DMABA-d10 NHS ester | Aldehyde,Ketones,Esters | Esters |
| MW0007115 | Fenfluramine | Benzene and substituted derivatives | Benzene and substituted derivatives |
| MW0007293 | Haloxyfop | Benzene and substituted derivatives | Benzene and substituted derivatives |
| MW0114584 | Heptaminol | Alcohol and amines | Alcohols |
| MW0110621 | Hexadecylamine | Alcohol and amines | Amines |
| MW0151442 | Ile-Gly-Val | Amino acid and Its metabolites | Small Peptide |
| MW0007392 | Irbesartan | Benzene and substituted derivatives | Benzene and substituted derivatives |
| MW0114733 | L-Gulose | Carbohydrates and Its metabolites | Sugars |
| MW0107803 | Leu-Ser | Amino acid and Its metabolites | Small Peptide |
| MW0108746 | N-(2,6-dimethylphenyl)piperidine-2-carboxamide | Heterocyclic compounds | Heterocyclic compounds |
| MW0008242 | N-(3-nitrophenyl)decanamide | Benzene and substituted derivatives | Benzene and substituted derivatives |
| MW0009030 | N-Desmethyl tapentadol | Benzene and substituted derivatives | Benzene and substituted derivatives |
| MW0055335 | N-octanoylsphingosine 1-phosphate | SL | Cer |
| MW0111202 | Nefopam | Heterocyclic compounds | Heterocyclic compounds |
| MW0108971 | O-benzyl-L-serine | Amino acid and Its metabolites | Amino acid derivatives |
| MW0109034 | palmyramide A | Alcohol and amines | Amines |
| MW0109079 | Perindopril | Heterocyclic compounds | Heterocyclic compounds |
| MW0116032 | Piperlonguminine | Heterocyclic compounds | Heterocyclic compounds |
| MW0109300 | Prilocaine | Alcohol and amines | Amines |
| MW0126261 | Propyphenazone | Aldehyde,Ketones,Esters | Ketones |
| MW0118880 | Pyrimidine-2-carboxylic acid | Organic acid and Its derivatives | Organic acid and Its derivatives |
| MW0009684 | Rivastigmine | Benzene and substituted derivatives | Benzene and substituted derivatives |
| MW0111278 | Scorpion | Heterocyclic compounds | Heterocyclic compounds |
| MW0009918 | Thonzylamine | Benzene and substituted derivatives | Phenolics |
| MW0157864 | Thr-Val-Leu-Arg | Amino acid and Its metabolites | Small Peptide |
| MW0127003 | Zileuton | Heterocyclic compounds | Heterocyclic compounds |
| LIPID-N-0010 | Ursocholic acid | ST | BA |
| LIPID-N-0014 | lithocholic acid-3-sulfate | ST | BA |
| LIPID-N-0015 | Glycocholic acid | ST | BA |
| LIPID-N-0058 | 5-iso PGF2VI | FA | Eicosanoid |
| LIPID-N-0061 | PGF1α | FA | Eicosanoid |
| LIPID-N-0062 | TxB3 | FA | Eicosanoid |
| LIPID-N-0063 | TXB2 | FA | Eicosanoid |
| LIPID-N-0067 | FFA(10:0) | FA | FFA |
| LIPID-N-0655 | LPA(16:3) | GP | LPA |
| LIPID-P-0004 | Carnitine C4:0 | FA | CAR |

**Supplementary Table 6 Metabolites in the yellow module in WGCNA**

| Index | Compounds | Class I | Class II |
| --- | --- | --- | --- |
| MEDN0226 | D-Erythronolactone | Aldehyde,Ketones,Esters | Esters |
| MEDP1814 | 2,5-Dimethyl-2,3-dihydrofuran-3-one | Heterocyclic compounds | Heterocyclic compounds |
| MEDP1212 | 1,3-Dicyclohexylurea | Alcohol and amines | Polyamines |
| MEDN1231 | 1-O-vanillyl-β-D-glucose | Benzene and substituted derivatives | Benzene and substituted derivatives |
| MEDP1485 | 2-(2-Benzothiazolylthio)ethanol | Alcohol and amines | Alcohols |
| MEDN0415 | 2-(Formylamino)Benzoic Acid | Benzene and substituted derivatives | Phenolic acids |
| MEDN2111 | 2-(Acetylamino)-2-deoxy-A-D-glucopyranose | Alcohol and amines | Amines |
| MEDP0545 | 2-Pyrrolidinone | Heterocyclic compounds | Heterocyclic compounds |
| MEDP0618*146 | Carnitine-2-methyl-C4 | FA | CAR |
| MEDN2009 | 2-Hydroxy-3-Methyl Butanoic Acid | Organic acid and Its derivatives | Organic acid and Its derivatives |
| MEDP2008 | 2-Hydroxy-4-(methylthio)butyric acid | Organic acid and Its derivatives | Organic acid and Its derivatives |
| MEDP1251 | 2-Mercaptobenzothiazole | Heterocyclic compounds | Heterocyclic compounds |
| MEDP1646 | Caldine | Alcohol and amines | Amines |
| MEDP2105 | 3,6,9,12,15,18,21-Heptaoxatricosane-21,23-diol | Benzene and substituted derivatives | Benzene and substituted derivatives |
| MEDP1898 | 3-Hydroxyphenylurea | Benzene and substituted derivatives | Benzene and substituted derivatives |
| MEDN0833 | 3-Dehydroshikimate | Organic acid and Its derivatives | Organic acid and Its derivatives |
| MEDN0410 | 4-Hydroxybenzaldehyde | Benzene and substituted derivatives | Benzene and substituted derivatives |
| MEDP0526 | 6-Dimethylaminopurine | Nucleotide and Its metabolites | Nucleotide and Its metabolites |
| MEDP2691 | 6-Methylthiopurine 5'-monophosphate ribonucleotide | Nucleotide and Its metabolites | Nucleotide and Its metabolites |
| MEDP0227*128 | D-Mannose | Carbohydrates and Its metabolites | Sugars |
| MEDP0224*128 | D-Fructose | Carbohydrates and Its metabolites | Sugars |
| MEDN0461 | D-Sedoheptuiose 7-Phosphate | Carbohydrates and Its metabolites | Phosphate sugars |
| MEDN1910 | Cys-Pro | Amino acid and Its metabolites | Small Peptide |
| MEDP1882 | Phe-Leu | Amino acid and Its metabolites | Small Peptide |
| MEDN1950 | Met-Asp | Amino acid and Its metabolites | Small Peptide |
| MEDN1559 | Pro-Gly | Amino acid and Its metabolites | Small Peptide |
| MEDP0321 | L-Dihydroorotic Acid | Organic acid and Its derivatives | Organic acid and Its derivatives |
| MEDP2478 | N-acetyl-D-phenylalanine | Amino acid and Its metabolites | Amino acid derivatives |
| MEDP2634 | Ethyl N-acetyl-L-tyrosinate | Amino acid and Its metabolites | Amino acid derivatives |
| MEDP0064 | N-Acetylcysteine | Amino acid and Its metabolites | Amino acid derivatives |
| MEDP0367 | γ-Aminobutyric Acid | Amino acid and Its metabolites | Amino acid derivatives |
| MEDP1860 | Glu-Leu | Amino acid and Its metabolites | Small Peptide |
| MEDN1532 | D-Galacturonic Acid | Carbohydrates and Its metabolites | Sugar acids |
| MEDP1749 | Cytidine 5'-diphosphate | Nucleotide and Its metabolites | Nucleotide and Its metabolites |
| MEDP2365 | Ala-Pro | Amino acid and Its metabolites | Small Peptide |
| MEDP2458 | Heptethylene-glycol | Alcohol and amines | Alcohols |
| MEDN2295 | Estradiol cypionate | Hormones and hormone related compounds | Hormones and hormone related compounds |
| MEDP0404 | Sarcosine | Amino acid and Its metabolites | Amino acids |
| MEDP0135*128 | Myoinositol | Carbohydrates and Its metabolites | Sugar alcohols |
| MEDN0499 | Argininosuccinic acid | Organic acid and Its derivatives | Organic acid and Its derivatives |
| MEDP0239 | L-Ascorbate | CoEnzyme and vitamins | CoEnzyme and vitamins |
| MEDP0892 | Pyrocatechol | Benzene and substituted derivatives | Phenolics |
| MEDP2068 | Hexaethylene-glycol | Alcohol and amines | Alcohols |
| MEDN0032 | Allantoin | Organic acid and Its derivatives | Organic acid and Its derivatives |
| MEDP1885 | Pro-Ile | Amino acid and Its metabolites | Small Peptide |
| MEDN0555 | Hydroxyphenyllactic acid | Organic acid and Its derivatives | Organic acid and Its derivatives |
| MEDP2753 | Azelaoyl PAF | FA | Others |
| MEDP1434 | Carnitine C4:DC | FA | CAR |
| MEDP2436 | Tetraethylene-glycol | Alcohol and amines | Alcohols |
| MEDP0692 | Triethylamine | Others | Hydrocarbon derivatives |
| MEDN1942 | Val-Thr | Amino acid and Its metabolites | Small Peptide |
| MW0049084 | (3S,3aS,6R,8aS)-3,8-Dimethyl-5-(propan-2-ylidene)-2,3,4,5,6,8a-hexahydro-1H-3a,6-epoxyazulen-6-ol | Alcohol and amines | Alcohols |
| MW0128633 | [3-(2H-1,3-benzodioxol-5-yl)-1-(4-methoxy-1-benzofuran-5-yl)-3-oxopropoxy]sulfonic acid | Organic acid and Its derivatives | Organic acid and Its derivatives |
| MW0128815 | [5-(3,5-dihydroxy-7-methoxy-4-oxo-4H-chromen-2-yl)-2-hydroxyphenyl]oxidanesulfonic acid | Organic acid and Its derivatives | Organic acid and Its derivatives |
| MW0059085 | 1-hexadecyl-2-(9Z-octadecenoyl)-sn-glycero-3-phosphoethanolamine | GP | PE-P |
| MW0142212 | 2-Amino-1,2-bis(p-chlorophenyl)ethanol | Heterocyclic compounds | Heterocyclic compounds |
| MW0123286 | 3-[(E)-3-(4-methoxyphenyl)prop-2-enoyl]-4-phenyl-1H-quinolin-2-one | Others | Medicine |
| MW0119987 | 4-(Methylnitrosamino)-1-(3-pyridyl-N-oxide)-1-butanol | Heterocyclic compounds | Heterocyclic compounds |
| MW0140438 | 4-(Phosphooxymethyl)-2-furancarboxaldehyde | Organic acid and Its derivatives | Organic acid and Its derivatives |
| MW0143282 | 4-Chlorochalcone | Aldehyde,Ketones,Esters | Ketones |
| MEDN1433 | 6-trans-12-epi Leukotriene B4 | FA | Oxidized lipids |
| MW0104968 | Beta-Tyrosine | Benzene and substituted derivatives | Benzene and substituted derivatives |
| MW0106119 | Carglumic acid | Organic acid and Its derivatives | Organic acid and Its derivatives |
| MW0159810 | Cyano-4-hydroxycinnamic acid | Alcohol and amines | Alcohols |
| MW0106796 | Dapt | Amino acid and Its metabolites | Amino acid derivatives |
| MW0161276 | Estra-1,3,5(10)-triene-3,17-diol | Alcohol and amines | Alcohols |
| MW0104081 | Eugenol sulfate | Organic acid and Its derivatives | Sulfonic acids |
| MW0007143 | Fenuron | Benzene and substituted derivatives | Benzene and substituted derivatives |
| MW0124123 | Flibanserin | Others | Medicine |
| MW0106717 | Formothion | Aldehyde,Ketones,Esters | Aldehydes |
| MW0114953 | Glcnac6P | Organic acid and Its derivatives | Organic acid and Its derivatives |
| MW0169331 | Isoproturon | Benzene and substituted derivatives | Benzene and substituted derivatives |
| MW0124521 | Italicene ether | Heterocyclic compounds | Heterocyclic compounds |
| MW0110123 | N2-Tryptophyllysine | Amino acid and Its metabolites | Small Peptide |
| MW0155422 | Phe4Cl-Asp-OH | Amino acid and Its metabolites | Small Peptide |
| MW0000400 | Piperolactam A | Alkaloids | Alkaloids |
| MW0126489 | Rutaecarpine | Heterocyclic compounds | Heterocyclic compounds |
| MW0009783 | Sulfachlorpyridazine | Benzene and substituted derivatives | Benzene and substituted derivatives |
| MW0110043 | Tris(2-carboxyethyl)phosphine hydrochloride | Organic acid and Its derivatives | Organic acid and Its derivatives |
| MW0194143 | (1S)-Deltamethrin | Benzene and substituted derivatives | Benzene and substituted derivatives |
| MW0012935 | (2r)-3-(Hexadecyloxy)-2-methoxypropyl 2-(trimethylammonio)ethyl phosphate | GP | PC |
| MW0142773 | (3-Acrylamidopropyl)trimethylammonium Chloride | Benzene and substituted derivatives | Benzene and substituted derivatives |
| MW0123400 | 1-[(2-Chloro-1,3-thiazol-5-yl)methyl]-2-methyl-3-nitroguanidine | Heterocyclic compounds | Pyridine and pyridine derivatives |
| MW0141090 | 1,2-Bis(chloromethoxy)ethane | Others | Others |
| MW0001300 | 1,2-Diphenylhydrazine | Benzene and substituted derivatives | Benzene and substituted derivatives |
| MW0118672 | 2-Methoxy-3,5-dimethylpyrimidine | Heterocyclic compounds | Heterocyclic compounds |
| MW0142036 | 2,4,6-Triaminotoluene | Benzene and substituted derivatives | Benzene and substituted derivatives |
| MW0004159 | 3,4-Dihydroxy-5-methoxybenzoic acid | Benzene and substituted derivatives | Benzene and substituted derivatives |
| MW0143303 | 4-Dodecylphenol | Benzene and substituted derivatives | Phenolics |
| MW0168630 | 5,7-Dichlorokynurenic acid | Organic acid and Its derivatives | Organic acid and Its derivatives |
| MW0144429 | AGELASINE | Heterocyclic compounds | Heterocyclic compounds |
| MW0144747 | Ala-Thr-Ile-Lys | Amino acid and Its metabolites | Small Peptide |
| MW0006049 | Anilazine | Benzene and substituted derivatives | Benzene and substituted derivatives |
| MW0105634 | Antanapeptin C | Benzene and substituted derivatives | Benzene and substituted derivatives |
| MW0145509 | Arg-Thr-Ala-Arg | Amino acid and Its metabolites | Small Peptide |
| MW0013528 | AS 1-1 | Alcohol and amines | Amines |
| MW0145998 | Asn-TyrMe-OH | Amino acid and Its metabolites | Small Peptide |
| MW0113948 | Azacitidine | Heterocyclic compounds | Heterocyclic compounds |
| MW0000168 | Capsaicin | Alkaloids | Alkaloids |
| MW0114068 | Chloromethyl methyl ether | Others | Others |
| MW0111487 | D-Lactaldehyde | Aldehyde,Ketones,Esters | Aldehydes |
| MW0114324 | d-Myo-inositol-1,4,5-triphosphate | Organic acid and Its derivatives | Organic acid and Its derivatives |
| MW0122332 | Dihydroneopterin phosphate | Organic acid and Its derivatives | Organic acid and Its derivatives |
| MW0006976 | Enilconazole | Benzene and substituted derivatives | Benzene and substituted derivatives |
| MW0106686 | Etidronic acid | Organic acid and Its derivatives | Organic acid and Its derivatives |
| MW0149253 | Flusulfamide | Benzene and substituted derivatives | Benzene and substituted derivatives |
| MW0149786 | Gln-Phe-Trp | Amino acid and Its metabolites | Small Peptide |
| MW0150398 | Gly-Leu-Arg-Val-Phe | Amino acid and Its metabolites | Small Peptide |
| MW0138459 | Isobavachalcone | Aldehyde,Ketones,Esters | Ketones |
| MW0153068 | Lys-Lys-Thr | Amino acid and Its metabolites | Small Peptide |
| MW0054761 | Menthol | Terpenoids | Terpenoids |
| MW0153665 | Met-Ser-Phe-Thr-Phe | Amino acid and Its metabolites | Small Peptide |
| MW0108345 | Monoethylglycinexylidide | Benzene and substituted derivatives | Benzene and substituted derivatives |
| MW0125223 | Moxonidine | Heterocyclic compounds | Heterocyclic compounds |
| MW0055397 | Notoginsenoside T1 | Terpenoids | Triterpene |
| MW0126295 | Pyrazin-2-carboxylic acid | Organic acid and Its derivatives | Organic acid and Its derivatives |
| MW0009796 | Sulfamethoxazole | Benzene and substituted derivatives | Benzene and substituted derivatives |
| MW0159883 | Sulfate | Others | Metal, Non-metal and related compounds |
| MW0158888 | Val-Arg-Ala-Glu | Amino acid and Its metabolites | Small Peptide |
| MW0159042 | Val-Ile-Leu-Asp | Amino acid and Its metabolites | Small Peptide |
| MW0159071 | Val-Leu-Lys-Val-Leu | Amino acid and Its metabolites | Small Peptide |
| LIPID-N-0032 | 15-oxoETE | FA | Eicosanoid |
| LIPID-N-0201 | LPS(22:6/0:0) | GP | LPS |

**Supplementary Table 7 Nine metabolites screened by LASSO and the random forest algorithm**

| Index | Compounds | Class I | Class II |
| --- | --- | --- | --- |
| MEDL01878 | Methyl dioxindole-3-acetate | Heterocyclic compounds | Indole and Its derivatives |
| MW0012989 | 1-Stearoyl-2-arachidonoyl-sn-glycero-3-phospho-(1'-myo-inositol) | GP | PI |
| MW0103492 | 4-Allylphenol | Benzene and substituted derivatives | Phenolics |
| MW0105082 | 3-Mercaptopyruvic acid | Organic acid and Its derivatives | Organic acid and Its derivatives |
| MW0133624 | 2-(2-Amino-3-methoxyphenyl)-4H-1-benzopyran-4-one | Benzene and substituted derivatives | Benzene and substituted derivatives |
| MW0149104 | Evoxanthidine | Heterocyclic compounds | Heterocyclic compounds |
| MW0152146 | Latia luciferin | Aldehyde,Ketones,Esters | Aldehydes |
| MW0155826 | Pro-Gln-Ala | Amino acid and Its metabolites | Small Peptide |
| MW0169290 | Icariin | Flavonoids | Flavonoid |
